# Strongly Emitting Folic Acid-Derived Carbon Nanodots for One- and Two-Photon Imaging of Lyotropic Myelin Figures

**DOI:** 10.1021/acsami.3c05656

**Published:** 2023-06-27

**Authors:** Dominika Benkowska-Biernacka, Sebastian G. Mucha, Lucyna Firlej, Filip Formalik, Jean-Louis Bantignies, Eric Anglaret, Marek Samoć, Katarzyna Matczyszyn

**Affiliations:** † Institute of Advanced Materials, Faculty of Chemistry, Wroclaw University of Science and Technology, 50-370 Wroclaw, Poland; ‡ Laboratoire Charles Coulomb (L2C), UMR5221, Université de Montpellier (CNRS), 34095 Montpellier, France; § Department of Physics and Astronomy, University of Missouri, Columbia, Missouri 65211, United States; ∥ Department of Chemical and Biological Engineering, Northwestern University, Evanston, Illinois 60208, United States; ⊥ Department of Micro, Nano, and Bioprocess Engineering, Faculty of Chemistry, Wroclaw University of Science and Technology, 50-370 Wroclaw, Poland

**Keywords:** carbon dots, nitrogen-doped nanomaterials, two-photon excited fluorescence, two-photon absorption, myelin figures, lipidic mesophases, lyotropic liquid crystals, two-photon microscopy

## Abstract

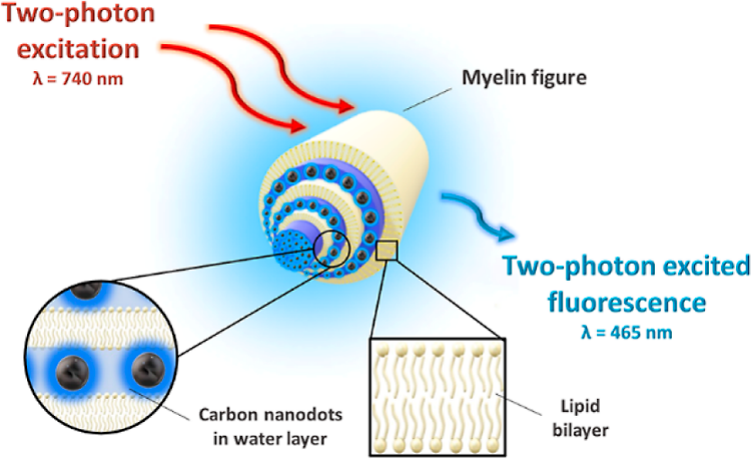

Non-invasive imaging of morphological changes in biologically relevant lipidic mesophases is essential for the understanding of membrane-mediated processes. However, its methodological aspects need to be further explored, with particular attention paid to the design of new excellent fluorescent probes. Here, we have demonstrated that bright and biocompatible folic acid-derived carbon nanodots (FA CNDs) may be successfully applied as fluorescent markers in one- and two-photon imaging of bioinspired myelin figures (MFs). Structural and optical properties of these new FA CNDs were first extensively characterized; they revealed remarkable fluorescence performance in linear and non-linear excitation regimes, justifying further applications. Then, confocal fluorescence microscopy and two-photon excited fluorescence microscopy were used to investigate a three-dimensional distribution of FA CNDs within the phospholipid-based MFs. Our results showed that FA CNDs are effective markers for imaging various forms and parts of multilamellar microstructures.

## 1 Introduction

Lipidic lyotropic liquid crystals have been extensively investigated in recent years, particularly in terms of their important functionalities exhibited in many biological structures.^1−4^ Among various applications, considerable attention was devoted to the potential use of mesophases as biomembrane mimetics for the development of novel tools for in vivo and in vitro imaging.^5−8^ In aqueous media, phospholipids spontaneously self-assemble and form bilayers of different spatial arrangements, including vesicles and cylindrical microstructures.^9−11^ Multilamellar tubes, composed of concentrically wrapped phospholipid bilayers alternating with aqueous layers,^12^ are known as myelin figures (MFs)^13^ to highlight their similarity to the myelin sheath promoting rapid nerve impulse propagation.^14,15^ As myelin is the target of several pathological pathways involved in incompletely understood disorders of the central nervous system,^16−18^ there is a need to develop sensitive and non-destructive probes to observe even subtle changes within this lipid-rich structure. In this context, combining biocompatible markers of nanometric size with a simplified model of the membrane could provide a new approach to studying phospholipid-based biologically relevant mesophases.^19^

To investigate the mechanism of MFs formation, many experimental methods have been used in the past: polarized light microscopy (PLM),^20^ digital holographic microscopy,^21^ wide-field fluorescence microscopy,^22^ and confocal fluorescence microscopy (CFM).^23^ The structural properties of MFs have also been extensively studied using X-ray scattering^12^ and electron microscopy.^24^ The use of non-invasive methods of MFs investigations that would enable in vivo bioimaging remains challenging. One of the most promising approaches is two-photon excited fluorescence microscopy (TPEFM).^25,26^ Two-photon excited fluorescence (TPEF) arises upon absorption of two low-energy photons (two-photon absorption; TPA) whose combined energy is sufficient to reach an excited state of the system. Typically, irradiation in the near-infrared (NIR) region (in the first biological window) is used. This NIR light is weakly absorbed and scattered by tissues and penetrates deeper into biological samples than the shorter wavelengths used for excitation of fluorescence through one-photon absorption.^27,28^

In most cases, the TPEFM imaging of biological systems is carried out not through autofluorescence (which is usually weak) but with the use of fluorescent markers. The design of new candidates for such an application needs to take into account physical, chemical, and biological aspects of the experiments such as the presence of effective TPA in the first biological-window wavelength range, high fluorescence quantum yield, and compatibility with aqueous media.^29,30^ Many fluorescent organic or organometallic dyes, macromolecular complexes, and inorganic and metallic nanoparticles fulfilling the above-mentioned criteria have been investigated. Recently, much attention has been directed at carbon dots (CDs), which seem to be excellent agents to use in biochemical assays.^30−36^

CDs are a versatile class of carbon nanomaterials with unique applicative properties: high biocompatibility, no cytotoxicity,^30,34,37^ facile and low-cost fabrication methods,^38−40^ long-term stability,^41^ and remarkable linear^42−44^ and non-linear optical properties.^30^ They also overcome many limitations of conventional fluorescent lipid probes (e.g., the Nile red dye), such as broad emission spectrum, small Stokes shift, and photobleaching.^45−47^

Hybrid systems consisting of phospholipids and highly fluorescent CDs have already been successfully applied to research on liposomes, e.g., to investigate the bilayer dynamics^19,48^ and formation of lipid rafts.^49^ A previous study on MFs formed in aqueous medium doped with latex microspheres has revealed that microparticles do not penetrate the lamellar phase; however, they can be applied as tracers to monitor the mechanism of the MFs’ growth.^50^ Accordingly, the use of CNDs with the above-mentioned properties may unlock new possibilities for doping and imaging of bioinspired cylindrical multilamellar structures.

Here, we demonstrate that newly developed folic acid-derived carbon nanodots (FA CNDs) can act as excellent markers in the MFs composed of zwitterionic phospholipids. We have first elaborated a facile and low-cost synthesis route to fabricate the FA CNDs and then studied their structural features in an aqueous environment using fluorescence in one-photon excitation (OPE) and two-photon excitation (TPE) regimes. Subsequently, we followed (using PLM) the formation of the phosphatidylcholines-based MFs doped with the as-prepared FA CNDs and showed that the presence of dopants does not interfere with lipid self-assembly into MFs. The distribution of the FA CNDs within the whole volume of the lamellar mesophase was also determined, using a combination of fluorescence microscopic and spectroscopic methods (including the TPEFM approach).

## 2 Results

### 2.1 Fabrication of FA CNDs

Folic acid (FA) is a non-fluorescent molecule which possesses a unique three-subunit structure rich in polar groups (oxygen- and nitrogen-like), and aromatic domains: heteroatom rings (pterin, PT), aryl amines (*p*-aminobenzoic acid, PABA), and amino acids (glutamic acids, GA). Hence, the FA is a promising precursor for facile synthesis of bright CNDs, expanding the group of new fluorescent nanomaterials; these CNDs can be dispersible in polar media (e.g., water) and interact with versatile biomolecular systems (including phospholipids) via electrostatic forces and hydrogen bonding.

**Figure 1:**
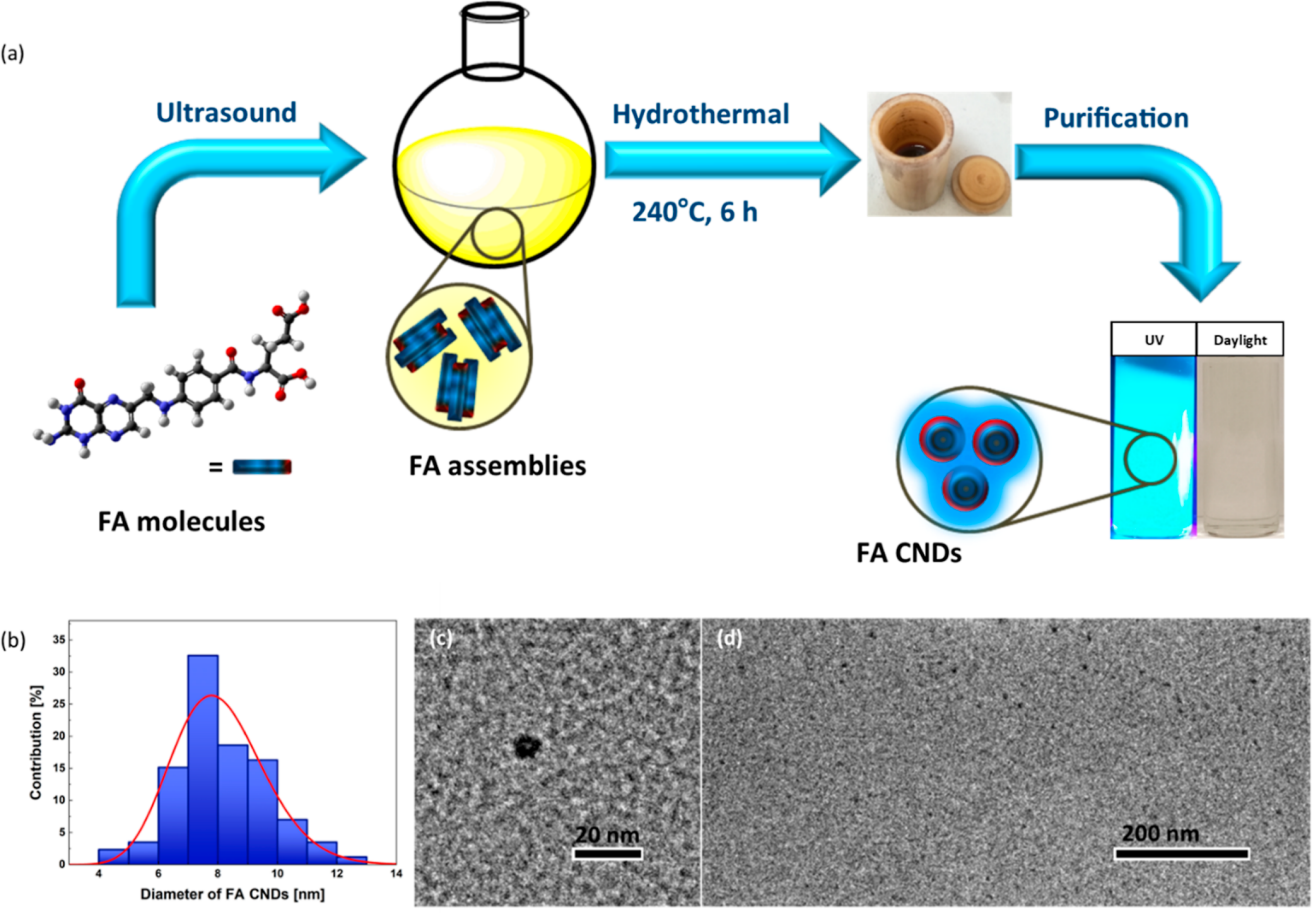
Synthesis of FA CNDs and their morphology. (a) Schematic illustration of the FA CNDs fabrication route: (i) ultrasound treatment, (ii) hydrothermal synthesis, and (iii) purification processes; the dispersion of FA CNDs was presented under daylight and UV irradiation (λ_exc._ = 365 nm); (b) size distribution of the FA CNDs with the Gaussian fitting curve; (c,d) zoomed HRTEM images of individual FA CNDs. The scale bars are (c) 20 and (d) 200 nm. More HRTEM images are available in Supporting Information.

Inspired by Liu’s synthesis procedure,^51^ we elaborated a new, two-pot engineering strategy for the fabrication of FA-based CNDs. It involves (i) ultrasonication of an aqueous FA mixture and (ii) hydrothermal synthesis (Figure 1a). In the first step, FA powder is dispersed in Milli-Q water (pH = 7.0) to reach the concentration of 15.1 mM and sonicated at room temperature. In such conditions, FA molecules agglomerate into new molecular and macromolecular forms. Such product is then subjected to hydrothermal treatment (at 240 °C), enabling the carbonization process and formation of FA CNDs. To separate the FA CNDs from the cocktail of other byproducts, the reactant is purified using silica column chromatography (with a mixture of chloroform and methanol as eluents). The FA CNDs are then filtrated and dried. The resulting dark yellow powder shows excellent dispersibility and long-term stability in aqueous media and simple alcohols (e.g., methanol). A more detailed procedure for synthesizing FA CNDs is provided in Materials and Methods.

The synthesis of FA CNDs is sensitive to the amount of precursor used. By varying the concentration of FA in Milli-Q water (3.63–60.4 mM), diverse synthesis yields were obtained (see Supporting Information, Figure S1). When the concentration of FA in the precursor mixture increases, the light scattering from the solution increases too, and the absorption bands gradually red-shift with respect to the aqueous solution of the FA (Figures S1 and S2). For instance, for C_FA_ = 15.1 mM (the optimal conditions of FA CNDs synthesis) a significant bathochromic shift is observed (the major absorption peak relocates from 280.0 to 319.5 nm). This indicates the formation of different macromolecular FA-based assemblies upon ultrasonication.

### 2.2 Structure Characterization of FA CNDs

The morphology and size distributions of FA CNDs were evaluated with HRTEM. As shown in Figures 1c,d, S3, and S4, the FA CNDs appear as well-dispersed nanoobjects with average diameters estimated to be 7.8 ± 2.6 nm. Although the size distribution is rather narrow, the aggregated forms are also observed (Figures S4 and S5).

The internal structure of FA CNDs was studied using IR, NMR, Raman, XPS, and EDX spectroscopies. To better identify the origin of each structural unit in FA CNDs, we consider the FA molecule and its three major sub-units (i.e., PT, PABA, and GA) as a reference (Figure S6).

**Figure 2:**
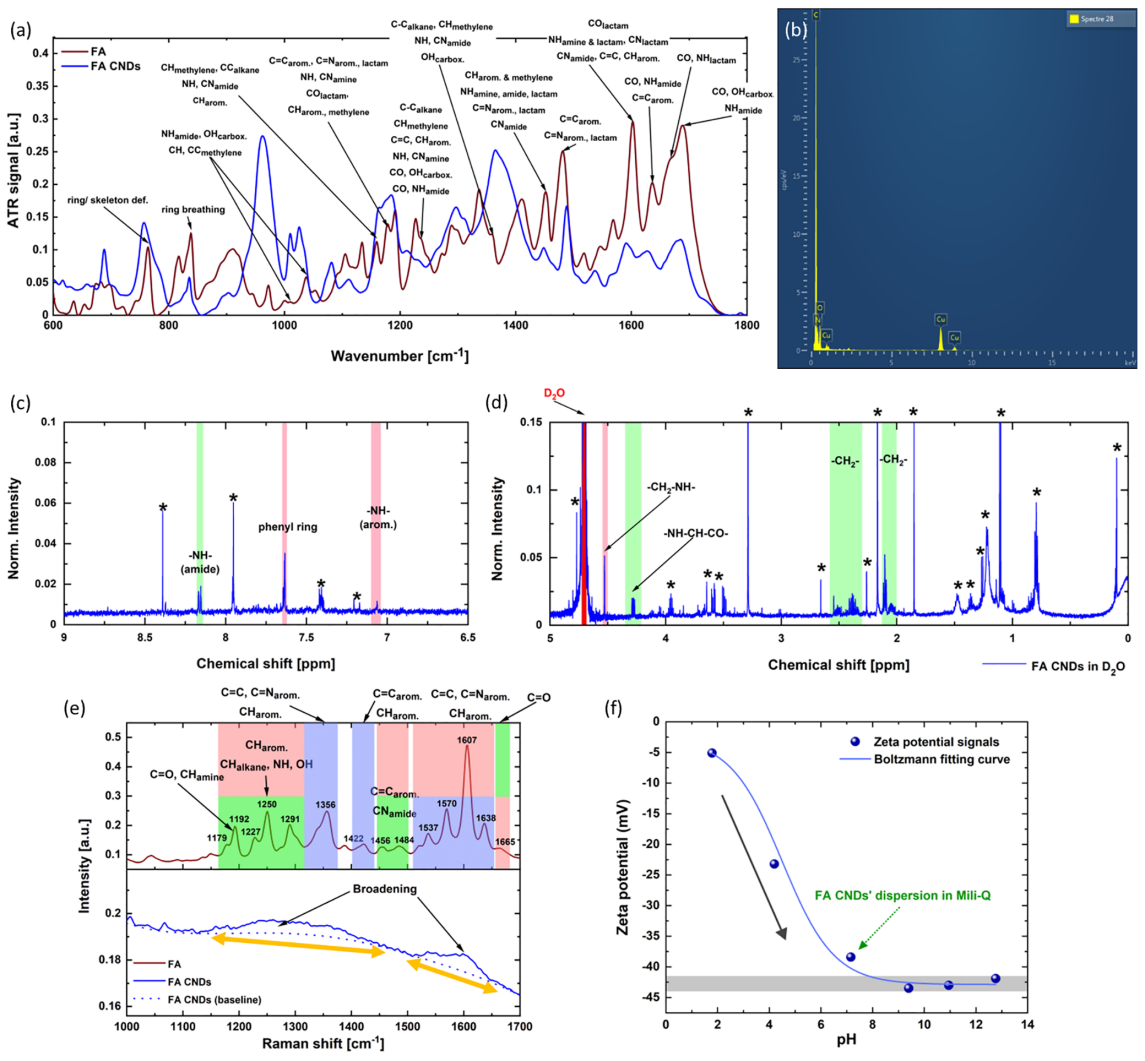
Structural properties of FA CNDs. (a) ATR-FTIR spectra of FA and the FA CNDs in the MIR region. The most significant chemical groups are assigned. (b) Representative EDX spectrum of FA CNDs. (c,d) Zoomed ^1^H NMR spectra of FA CNDs in ; the signal of D_2_O was marked with red color. Each NMR peak was normalized with respect to the strongest one located at 3.3 ppm (see Supporting Information for the full-range ^1^H NMR survey). The NMR signals common for FA CNDs and FA are assigned to particular chemical moieties in one of the three major sub-units of the molecular precursor (PT—blue, PABA—red, and GA—green). New peaks emerging in FA CNDs spectra are indicated with stars. (e) Raman spectra of FA and the FA CNDs. The most crucial peaks of FA are described (the position, the vibration mode, and structural origins: PT—pterin, PABA—*p*-aminobenzoic acid, GA—glutamic acid). Two significant changes in the Raman spectrum of FA CNDs (the spectral broadening and intensity lowering) are indicated with arrows. (f) Evolution of the zeta potential as a function of the pH value of the dispersion. The relation was fitted with the Boltzmann equation. The decreasing trend is indicated with a gray arrow, while the bold gray line corresponds to the plateau level of the zeta potential. The zeta potential signal of the non-titrated dispersion of FA CNDs is marked with a green arrow.

The basic chemical sub-groups of FA CNDs were identified using IR spectroscopy in the FIR and MIR regions.^52,53^ First, the vibrational spectrum of the FA molecule was analyzed using both calculated and experimental IR signals (Figures S7 and S8 and Tables S1–S3). Then, the IR spectrum of FA was compared with that of FA CNDs (Figures 2a and S9). FA CNDs are rich in polar groups (carboxylic and amide moieties); the IR signals appear at 1690 cm^–1^ (e.g., CO_stretching_, NH_bending_, and OH_bending_), at 1600 cm^–1^ (e.g., NH_bending_ and CO_bending_), at 1360 cm^–1^ (e.g., NH_bending_), and at 1240 cm^–1^ (e.g., CO_bending_). The IR components at 1600 cm^–1^ (e.g., C=C_stretching_ and CH_bending_), at 1480 cm^–1^ (e.g., C=N/C=C_stretchings_), and at 1450 cm^–1^ (e.g., C=C/C=N_stretchings_ and CH_bending_) can be assigned to the residual aromatic unit of the FA precursor. The IR peaks in the range of 2870–2980 cm^–1^, characteristic for stretching vibration (CH_stretching_), reveal the presence of sp^3^-hybridized carbon atoms in aliphatic chains. All of the above-mentioned signals appear at similar frequencies in both FA CNDs and FA molecules (Figures 2a and S9). The essential differences are found in the vibration regions related to inter- and intramolecular interactions which involve the polar sub-units. As illustrated in Figures 2a and S9, the IR signal of hydroxyl (OH) and amine (NH) stretching modes evolves from the broad band in the 2300–3700 cm^–1^ range (FA) to a narrower one (ca. 2700–3600 cm^–1^, FA CNDs). Besides, sharp high-frequency IR components appearing in the FA spectrum at 3400, 3530, and 3550 cm^–1^ (hydrogen-bond-free NH and OH stretching modes) become broader and are downshifted in the FA CNDs.

We conclude that the OH and NH moieties of nanodots form new inter- and intramolecular hydrogen bonds as compared to FA molecules. As expected,^54^ some carbonyl groups of FA CNDs are also involved in new hydrogen-bonded assemblies (Figures 2a and S9): the IR bands of FA CNDs at 1630 and 1590 cm^–1^ (dominated by the CO_stretching_) are downshifted when compared to the signals of FA molecules (Table S1). The rearrangement of a hydrogen-bonding network in FA CNDs can be also deduced from the analysis of the FIR region of the spectrum. A new intense and broad band emerges at 166 cm^–1^ (FA CNDs, Figure S9), masking the peaks of torsional vibrations located below 230 cm^–1^ (Table S3). This indicates a collective character of hydrogen bonds in carbon nanodots.^55,56^

The structural characteristics of FA CNDs were also explored with ^1^H and ^13^C NMR; then hydrogen–carbon (heteronuclear single quantum coherence, HSQC NMR) analysis^57−63^ was performed. The ^1^H NMR spectrum of FA CNDs is complex, especially in the upfield region (0–5 ppm), as shown in Figures 2d and S10. The peaks correspond to the protons from a variety of aliphatic carbon forms, both non-polar (e.g., at 1.1, 1.9, and 2.2 ppm, carbon core) and polar (e.g., amine, amide, or carboxyl groups; 3.3 and 4.5 ppm). The less intense signals in the downfield range (Figures 2c and S10) indicate the (minor) presence of aromatic domains (e.g., at 7.6 and 8.4 ppm) and amine units (at 7.1 and 8.2 ppm). The abundance of the aliphatic carbon form in FA CNDs is confirmed by the ^13^C NMR spectrum (Figure S11). Intense peaks at 23.8 and 30.3 ppm originate from sp^3^-hybridized carbon atoms adjacent to non-polar groups. The signals at 49.0 and 64.3 ppm indicate the proximity of polar moieties. Weak signals at 187.0 and 188.3 ppm come from the carbonyl group in carboxylic acids or amides. The signature of aromatic domains of FA CNDs is undetectable. It should be stressed that the majority of peaks cannot be attributed to the precursor molecule. Therefore, in agreement with the rotational-vibrational analysis, the NMR results confirm the significant evolution of the FA molecules during the formation of FA CNDs.

We also compared the Raman spectra of a FA molecule and FA CNDs (Figures 2e and S13).^52,64,65^ As depicted in Figure S13, many vibration modes observed in FA (e.g., at 1250, 1360, and 1610 cm^–1^) disappear in FA CNDs. The narrow Raman bands in the ranges of 1100–1450 and 1510–1650 cm^–1^ significantly broaden and become close to the D and G bands expected for nanocrystalline and amorphous carbon, respectively.^66^ These findings reveal that the structure of FA CNDs is more complex than that of the precursor and consists of heterogeneously distributed sp^2^- and sp^3^-hybridized carbon domains.

The chemical composition of FA CNDs was analyzed using XPS and EDX assays. FA CNDs contain both oxygen and nitrogen (Figures 2b, S14, and S15 and Table S5) but in a proportion that differs from that of the FA precursor. The content of nitrogen is lower (atomic percentage of ∼30% versus 54% in FA molecules). It agrees with the previously observed decreasing content of nitrogen-rich PT sub-unit and its derivatives in the internal structure of a nanodot.

The external charge of FA CNDs was studied by monitoring the evolution of zeta potential (ζ) as a function of the pH (Figures 2f, S16, and S17). In acidic media, the highest ζ appears at pH = 1.79 (∼−5 mV) and tends to decrease as the pH grows. At the pH = 7.2, the ζ parameter is −38 mV. The ζ decreases to −43 mV (at pH = 9.40) and then reaches a plateau with nearly similar values under alkaline conditions.

These findings reveal that the FA CNDs are negatively charged in the neutral aqueous environment. The sharp reduction of the negative charge of nanodots in acidic media is consistent with the presence of carboxyl groups. When the pH increases, they tend to be deprotonated and appear in an anionic form at the neutral pH. The minor decrease at the basic pH indicates the deprotonation of other protic polar groups (i.e., hydroxyl moieties).

Overall, the structural investigations indicate that the internal structure of FA CNDs evolves from that of a relatively small molecule to a nanodot with a more complex design consisting of highly ordered sp^2^-hybridized domains embedded within the prevalent sp^3^-carbon matrix. New polar and non-polar groups appear; although some FA residues (mainly the GA- and PABA-like ones) are still present.

### 2.3 One-Photon Excited Fluorescence of FA CNDs

**Figure 3:**
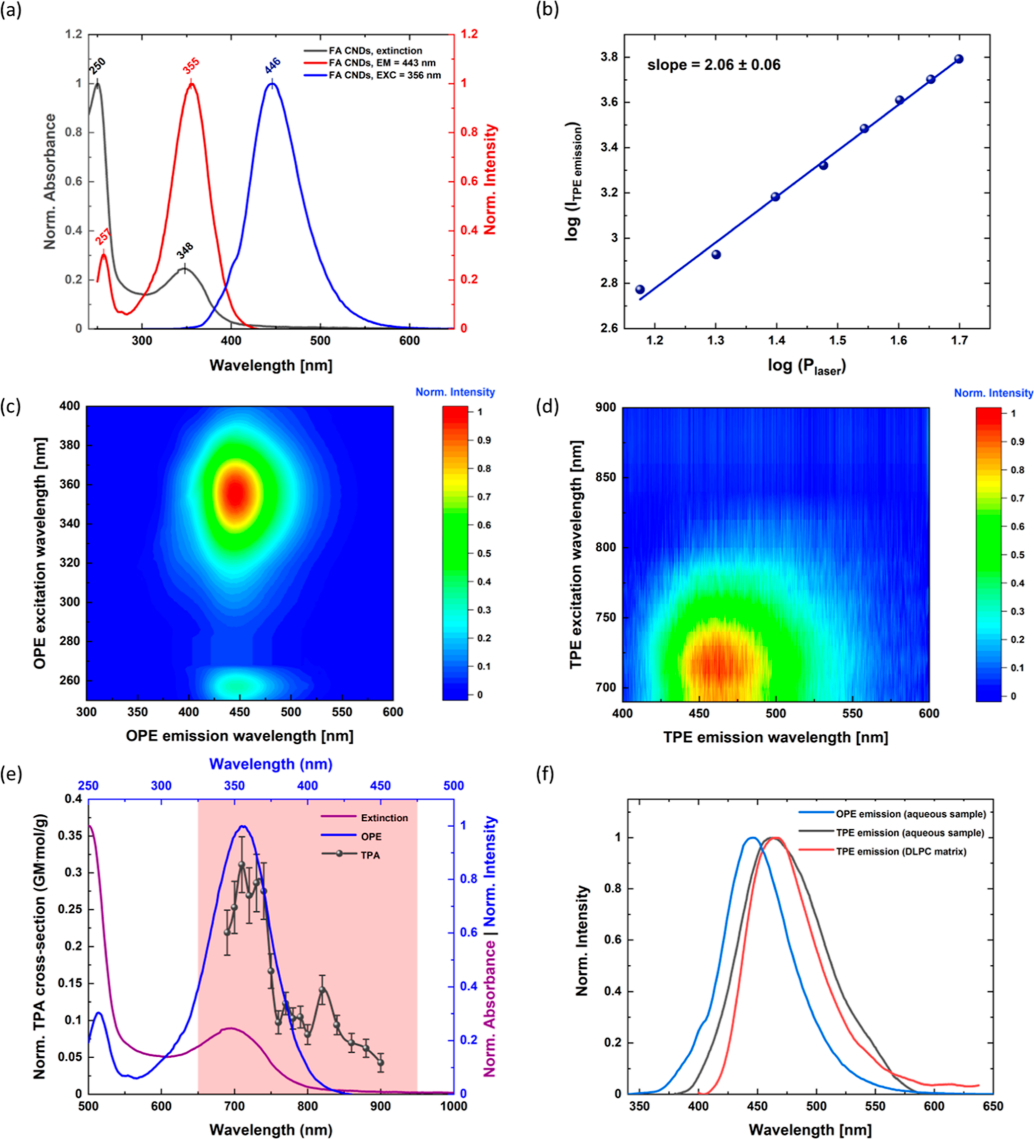
Linear and non-linear optical performance of the FA CNDs. (a) Left axis: normalized extinction spectra of the FA CNDs. (black). Right axis: OPE (red) and OPEF (blue) spectra of the FA CNDs. (b) Log(*I*_T__PE emission_) vs log(*P*_laser_) plots at λ_exc._ = 692 nm. (c) One-photon excitation-emission map of the FA CNDs. (d) Two-photon excitation-emission map of the FA CNDs. The TPE wavelength intervals are 10 nm (690–800 nm) and 20 nm (800–900 nm). (e) Left axis: OPA (violet), and OPE (blue) spectra of FA CNDs. Right axis: TPA (gray) spectra of FA CNDs. The red area indicates the first biological window (650–950 nm). (f) OPEF (blue) and TPEF spectra of the FA CNDs in an aqueous suspension (gray) and DLPC matrix (red).

Figures 3a and S18 show UV–Vis–NIR extinction spectra of FA and FA CNDs dispersed in water (pH = 7.0). An aqueous solution of FA molecules exhibits two characteristic OPA peaks, centered at 280 and 344 nm. They can be assigned to the π–π* (S_0_–S_*n*_) and *n*–π* (S_0_–S_1_) electronic transitions within PT and PABA residues.^67,68^ In the case of FA CNDs, the strong and sharp absorption band at 250 nm and a weak absorption peak centered at 348 nm may indicate the presence of fused PT- and PABA-based aromatic domains (however, after some structural rearrangements^68^). The FA CNDs themselves show two peaks in the OPE spectra: one intense (at 355 nm) and one weaker (at 257 nm). The differences between OPA and OPE signals suggest that the excitation processes are most efficient when a longer excitation wavelength is used. Similar behavior has been observed for FA-like molecular fluorophores (Figure S18) and other FA-derived nanostructures.^69−71^ Upon excitation in the wide wavelength range, the FA CNDs show an intense, symmetric, and stable one-photon-excited fluorescence (OPEF) band at 446 nm (Figure 3a,c). A sharp OPEF peak (with full-width at half-maximum estimated to be 65 nm) in the blue region evidences almost monochromatic fluorescence color, as observed in an aqueous dispersion of FA CNDs exposed to UV light (Figure 1a). It is noteworthy that the FA CNDs have a large Stokes’ shift (ca. 98 nm) which reduces the risk of undesired re-absorption processes.

Although the position of OPEF peaks of the FA CNDs and the FA precursors are very similar (λ_em.max_ (FA CNDs) = 446 nm and λ_em.max_ (FA) = 441 nm), their fluorescence efficiencies (expressed as the absolute fluorescence quantum yield, FQY) vary significantly. The FQY value of the aqueous suspension of FA CNDs excited with a laser pulse at 377 nm is equal to 54.1%, more than 2 orders of magnitude larger than for FA molecules.^69^ This performance is excellent compared to a series of other blue-emitting CDs^70,71^ or inorganic semiconductor nanoparticles in aqueous dispersions and agrees with the data reported in the literature for most of the fluorescent FA-based CDs and organic fluorophores (Table S7).^51,72−74^

To unravel the fluorescence dynamics, the OPEF decays of the FA CNDs were measured using the time-correlated single-photon counting method (TCSPC; λ_em._ = 446 nm, λ_exc._ = 377 nm). The as-obtained curves of fluorescence decay (Figure S20) can be adjusted by assuming two components: a dominant one (∼86%), with a long lifetime (of 7.7 ns), and a shorter one (2.0 ns). The average fluorescence lifetime (⟨τ⟩) of ∼7.5 ns (Table S8) indicates that the fluorescence of FA CNDs is relatively long-living. It is an essential property of all FA-derived nanomaterials^51,72^ and other nitrogen-doped CDs in aqueous dispersions.^75−77^

The pH-induced evolution of the fluorescence of FA CNDs was monitored using the OPEF spectra. The FA CNDs exhibit strong fluorescence over the whole pH range studied here (i.e., from 3.8 to 9.0; Figure S19). No spectral shift in the OPEF band was observed. The intensity of the OPEF signal, measured with respect to the intensity at neutral (pH = 7.0) conditions, gradually increases with the pH of the environment (*I*/*I*_neutral pH_ ∼ 0.85 in acidic conditions, pH = 3.8) and (*I*/*I*_neutral pH_ ∼ 1.10 in alkalic conditions, pH = 8.7). In fact, FA CNDs also maintain efficient fluorescence in acidic and basic environments, as evidenced by high FQY values: 36.6% (at pH = 4.2) and 46.9% (at pH = 8.7). The above trend may be explained by the internal structure of the FA CNDs, rich in diverse polar moieties (i.e., carboxyl, hydroxyl, and amino groups). The absence of sharp fluorescence decrease in the acidic dispersion of the FA CNDs suggests that carboxyl groups appear in deprotonated forms, creating the negatively charged “protective shell”.^30,78^ This conclusion is consistent with the negative ζ and its lowering trend: values decrease from −5 to −38 mV (in the pH range of 1.8–7.2). Meanwhile, under neutral and basic conditions, the hydroxyl and amino sub-units tend to be deprotonated gradually (the ζ decreases slightly), resulting in the final anionic form. A negative charge of a single dot becomes stronger.^39^ As a result, the overall fluorescence response of the FA CNDs increases.

### 2.4 Two-Photon Excited Fluorescence of FA CNDs

The fluorescence of FA CNDs is also observed in the non-linear optical regime. Excitation with femtosecond laser pulses in the 690–800 nm region (the first biological window)^27^ produces intense blue emission with no excitation-dependent trend (Figure 3d). The double logarithmic plot (log(*I*_TPE emission_) vs log(*P*_laser_), Figure 3b) illustrates that the intensity of the fluorescence signal increases roughly quadratically as a function of the input laser power (the slope value is equal to 2.06 ± 0.06). It proves that the observed TPE process does not involve OPA.^79^

The TPA performance of the FA CNDs can be quantified by giving a microscopic TPA cross section of an individual absorbing entity [σ_TPA_, in Goeppert-Mayer (GM) units: 1 GM = 10^–50^ cm^4^ s]. The σ_TPA_ values and the fluorescence quantum yield φ determine together the brightness of TPEF from a single particle. However, they do not allow an easy comparison of the TPA of materials (and nanomaterials) of different chemical composition and/or structure. The scaling of the σ_TPA_ values by a relevant structural parameter, such as molar mass (*M*) or molar volume, is necessary to obtain an appropriate merit factor σ_TPA_/*M*.^80−83^

In the case of the FA CNDs, the σ_TPA_/*M* values were determined using the TPE luminescence method, taking a solution of perylene in dichloromethane as a TPA reference.^80,84^ The obtained σ_TPA_/*M* merit factor at 710 nm is equal to ∼0.31 GM·mol/g (Figure 3e). This result is comparable with the σ_TPA_/*M* values observed for the most effective two-photon absorbers (including organic dyes,^84^ macromolecular complexes,^82,85^ or inorganic semiconductor nanomaterials^86−89^) in the red and NIR windows (Figure S21). The advantage of FA CNDs is their capacity to disperse in water, whereas most of the reported compounds require either hydrophobic media (e.g., toluene or dichloromethane) or contain toxic heavy metals (e.g., Cd, In, or Ru) in their internal structures.

We also estimated the normalized two-photon brightness, expressed as the combination of σ_TPA_/*M* and φ parameters (φ·σ_TPA_/*M*). We conclude that the TPE performance of FA CNDs is excellent with respect to aqueous solutions of blue-emitting two-photon dyes reported so far (Figure S22)^79,90,91^ and suitable for bioimaging.

We also note that, in general, the one- and two-photon absorption processes do not necessarily lead to the same excited states (e.g., for symmetry reasons). In the case of FA CNDs, the TPA maximum appears at ca. 710 nm (the doubled wavelength of the OPA absorption peak), indicating that both types of electronic transitions promote the CNDs to excited states with the same energy (Figure 3e). It should be noted that in some CDs the TPE bands are blue-shifted with respect to the doubled-wavelength position of their OPA spectra.^92,93^ The TPE fluorescence band of the FA CNDs is red-shifted (by 17 nm) with respect to their OPEF spectra (Figure 3f). Such a feature was reported for a variety of fluorescent species containing polymeric sub-structures, including other CNDs,^93^ polymer dots (PDs),^30^ peptide aggregate forms (i.e., amyloids),^94^ etc.

### 2.5 Photostability

In addition to the excellent fluorescence performance, the potential fluorescent biomarker must be photostable. To evaluate the photostability of FA CNDs, the evolution of their OPEF spectra was monitored upon high-power illumination with UV light (λ_exc._ = 365 nm) and the NIR laser beam (λ_exc._ = 740 nm). It should be noted that these wavelengths lie within the intense OPA and TPA bands of FA CNDs, respectively. As shown in Figure S23, the OPEF intensity remains almost unchanged upon long-term (60 min) and continuous irradiation; they reach 99 and 93% of the initial values, respectively. This indicates the remarkable resistance of FA CNDs to photobleaching under illumination conditions, more destructive for FA CNDs than those used in the experiments.

### 2.6 Formation of MFs

The excellent OPEF and TPEF performance of the FA CNDs inspired us to explore their potential as fluorescence probes in the bioimaging of lipid-based MFs. Such elongated microstructures composed of phosphatidylcholines, one of the most abundant glycerophospholipids in cell membranes,^95^ constitute a simplified model of a biological entity. The MFs, made of 1,2-dilauroyl-*sn*-glycero-3-phosphocholine (DLPC), were prepared following the contact method reported in the literature,^13^ except that the aqueous phase was doped with the FA CNDs. Consequently, after the hydration of the dried droplet of DLPC, the lipid tubes doped with CNDs began to grow at the lipid–water interface. The elongated multilamellar structures were formed parallel to the glass substrate and tended to grow in the direction opposite to the lipid film, i.e., toward the aqueous phase.

**Figure 4:**
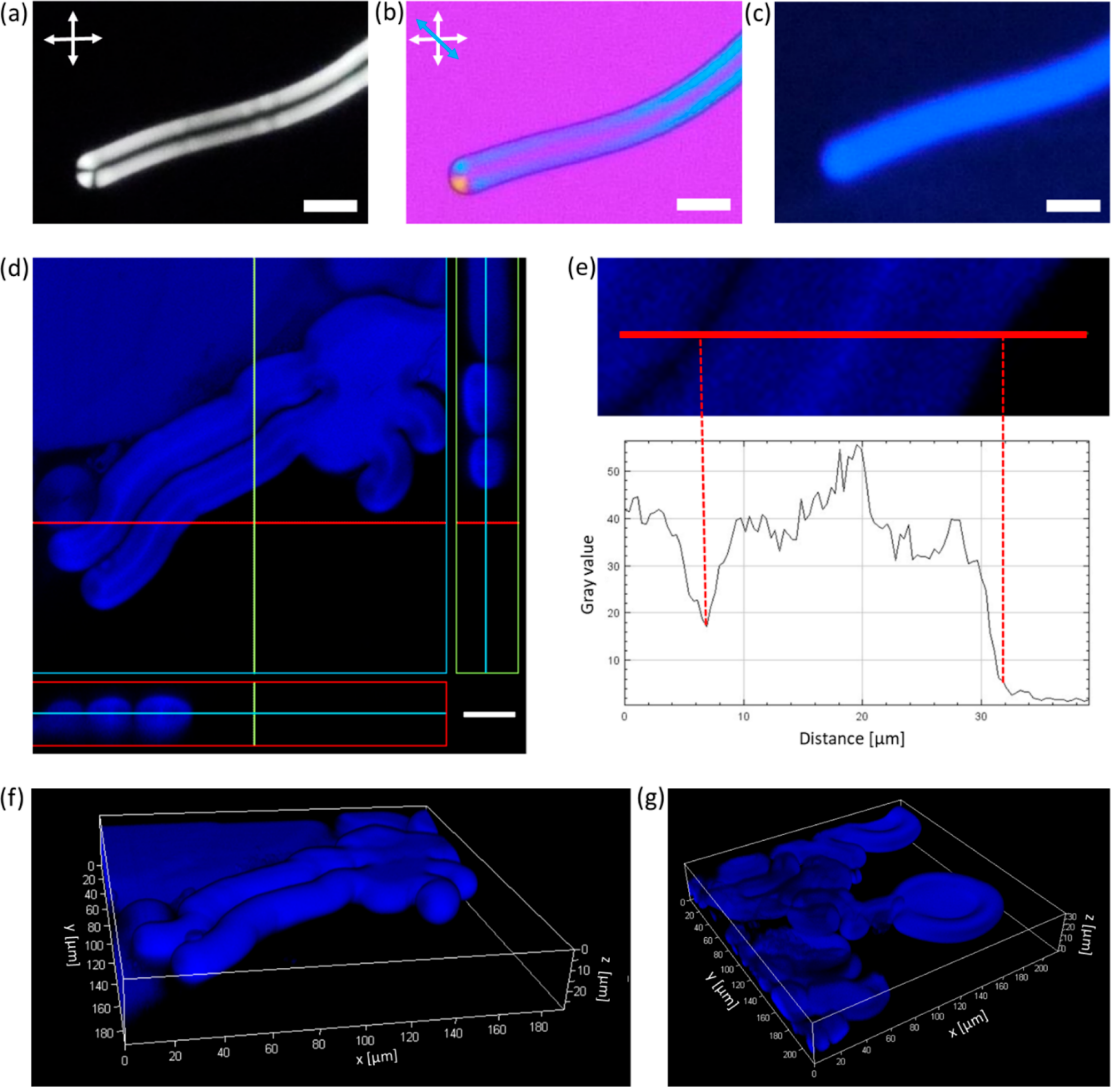
Polarized light and confocal fluorescence imaging of the MFs doped with the FA CNDs. (a,b) Representative polarized light optical images of the MF doped with the FA CNDs. White and blue arrows depict the orientation of the polarizers and the slow axis of the full-wavelength retardation plate, respectively. (c) Fluorescence image of the same area of the sample (λ_exc._ = 360 nm). (d) CFM image of the MFs with cross-sectional views taken along red and yellow lines in the planes perpendicular to the XY plane (blue square). (e) Line profile of pixel intensity taken along the red line of the CFM image shown above. (f,g) 3D reconstructions of CFM images show (f) straight and (g) oval-shaped MFs (λ_exc._ = 405 nm). The projections of the structures were obtained from *z*-stacks of 40 and 65 layers, respectively. The scale bars represent 10 μm in (a–c) and 25 μm in (d).

To gain better insight into the formation of the MFs in the presence of water dispersion of the FA CNDs in the liquid crystalline cell and evaluate their quality, PLM was used. The characteristic texture of MFs emerged in the doped sample (Figure 4a). The optical anisotropy of the MFs, observed between crossed polarizers as bright lines, results from the arrangement of rod-like DLPC molecules. Inserting a full-wavelength retardation plate into the optical path enables the determination of the lipid positions within the structure. The blue and yellow colors reflect the orientation of the long axis of phospholipids in the multilamellar structure at 0 or 90° with respect to the slow axis of the retardation plate. We verified that hydrocarbon chains of DLPC in the MFs’ walls are directed perpendicularly to the water core, around which bilayers are concentrically wrapped (Figure 4b). These results are in line with those obtained for pristine MFs (Figure S24).

The growth of representative MF doped with the FA CNDs was monitored by taking micrographs with a 5 s time interval. Changes in the lengths of the MF are shown in Figure S25. The fast growth rate within the first 50 s after hydration slows down considerably when the structure elongates. The diameters of the tubes remain unchanged during the MFs’ growth. Similar behavior was observed for the samples formed in the absence of CNDs.^96,97^ In addition to the straight and slightly bent cylindrical structures characteristic for both hybrid materials and dopant-free samples, looped and oval shapes were also observed (Figure S26). We conclude that adding the FA CNDs does not affect the formation of the specific multilamellar microstructures.

### 2.7 One-Photon Excited Fluorescence Imaging of MFs

To localize the FA CNDs in the sample, wide-field fluorescence microscopy was applied. Upon excitation at 360 nm, bright blue tubes were observed on the darker background (Figure 4c), indicating that the FA CNDs were well distributed along the phospholipid-based MFs. The FA CNDs do not only enter lamellar mesophase but also accumulate in it; the fluorescence signal arises mainly from the MFs, although some nanoparticles are also present in the entire field of the photograph. The fluorescence intensity appears to be homogeneous on both the edge of the lipid droplet and the external structures. It proves that the FA CNDs penetrate the MFs and not merely trap or aggregate at their periphery.

To verify the possibility of applying the FA CNDs as effective markers of the aqueous phase in phospholipid-based structures, we compared their use with fluorescein, which is commonly used as a water tracer. As shown in Figure S27, the background signal arising from fluorescein is intense, while the signal from the tubes is negligible. These findings imply that CNDs, providing a distinct contrast between the mesophase and the surrounding medium, perform better as a fluorescent probe of the aqueous phase in the MFs.

To further investigate the cross-sectional view of the MFs, CFM was used. The false-color image shown in Figure 4d reveals blue emission collected in the wavelength range of 410–480 nm (upon excitation at 405 nm), which is depicted on a black background. The high-resolution image provides sufficient contrast to distinguish different parts of the multilamellar lipid tube. The horizontal pixel intensity profile taken across the short axis of the MFs (Figure 4e) indicates that the highest intensity of fluorescence comes from the center of the MF. The intensity decreases by a factor of ∼1.4 on both sides of the most intensely colored region and drops sharply at the periphery of the bright part of the image. It confirms that although the largest quantity of dopants is localized in the water channel passing through the MFs, a considerable amount of them is also confined between lipid bilayers surrounding this core.

The samples were also scanned along the *z*-axis, with a small height step starting from the surface of the glass substrate and ending at the top of the MFs. Previous confocal studies^20,98^ indicated that lipid tubes growing from the preformed multilamellar structures show hollow cross-sectional views. Here, the lipid-free volume going through the structure contained abundant luminescent dopants. The orthogonal views in which cut-through sections are marked (Figure 4d) confirm that the strong fluorescence signal comes from the center of the MFs. For reference, the additional experiment under the same setup conditions was conducted on a sample without the FA CNDs. As shown in Figure S28, there was no fluorescence signal from the sample with the pure DLPC.

Rich information on the external surface of the multilamellar microstructure is provided by three-dimension (3D) projections made from *z*-stacks comprising 40 (Figure 4f) and 65 (Figure 4g) optical slices. The volume rendering visualization of differently shaped MFs enabled the observation of fine structure details, such as folds and concavities. The 3D reconstructions show that straight parts of the MFs are cylinder-shaped along the entire length, while the oval-shaped (resulting from the pressure reduction inside the MFs^13^) are collapsed ones.

### 2.8 Two-Photon Excited Fluorescence Imaging of MFs

**Figure 5:**
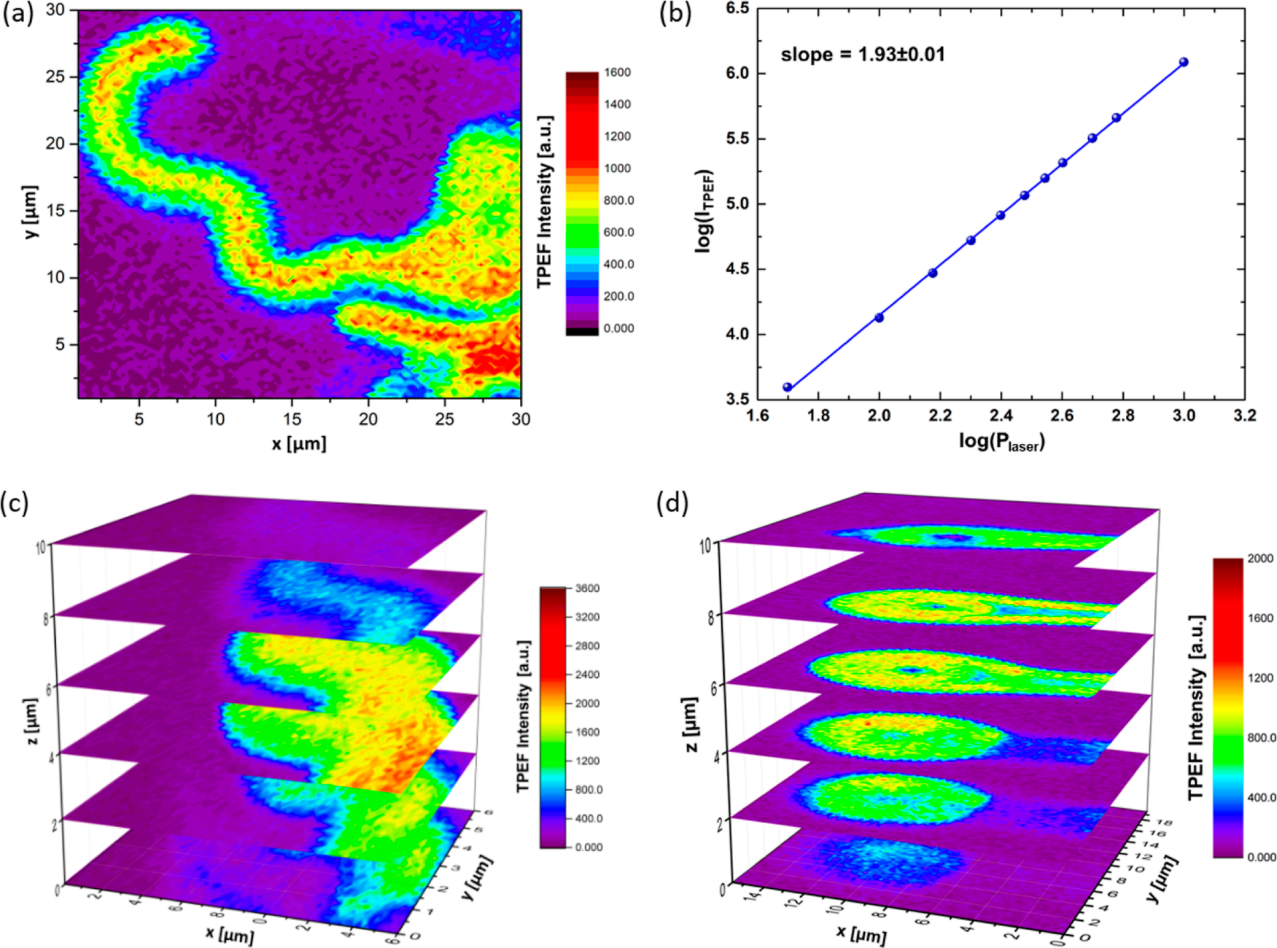
TPEF imaging of MFs doped with the FA CNDs. (a) 2D contour map of the TPEF intensity of the MFs doped with the FA CNDs. (b) Log(*I*_TPE__emission_) vs log(*P*_laser_) plots. (c,d) Stacked 2D contour maps of TPEF intensity at different *z*-positions from (c) straight and (d) oval MFs doped with the FA CNDs. The raster scans were performed along the *z*-axis with the step of 2 μm. The average power of the incident laser beam was 75 μW (λ_exc._ = 740 nm). The color code represents the emission intensity.

The response of pristine and FA CNDs-doped MFs to the excitation in the first biological window was analyzed using multiphoton microscopy. The liquid crystalline cell was placed upside down on the nanopositioning piezo stage (Figure S29), and a femtosecond laser beam with λ_exc._ = 740 nm was used as excitation. Then, the representative multilayered structures were scanned in the *XY* plane to detect fluorescence responses. The TPEF intensity map (Figure 5a) shows strong emission from lamellar mesophase doped with the FA CNDs. Weaker signals collected from the surrounding aqueous phase can be attributed to the emission from nanostructures dispersed homogeneously around the MFs. The emission intensity from the water core along the entire length of the MF is twice as high as the emission signal from its multilayered walls. The emission intensity of reference MFs made of pure DLPC was negligible compared to those observed in FA CNDs-doped MFs. The TPEFM images of the pristine sample were obtained using two and four times higher power of incident laser beam than that applied in experiments on the MFs containing fluorescent dopants (Figure S30); even so, sample damage was not observed.

To further explore the emission spectrum of the FA CNDs and the location of the dopant within the lipid matrix, a multi-photon microscopy experiment was carried out on an MF with a diameter two times larger than the average value observed in the samples. The fluorescence response was detected in the range of 400–700 nm, with the strongest emission signal (characteristic for FA CNDs) appearing at 465 nm. The highest fluorescence intensity was found along a line delimiting the inner water channel (Figure S31a). It suggests that the FA CNDs accumulate at the interface between the water core and the first lipid bilayers. This statement is supported by TPEF results showing lower emission intensity at the center of the water core than at its edge (Figure S31b). The TPEF spectrum is red-shifted with respect to the TPEF spectrum in an aqueous suspension (Figure 3f). It indicates that the FA CNDs remain fluorescent when diffusing from aqueous media into the MFs. The slope of log(I_T__PE emission_) vs log(*P*_la__ser_) of 1.93 ± 0.01 (Figure 5b) confirms that the fluorescence originates from the TPE process (as already observed for the FA CNDs dispersed in water, Figure 3b).

To verify the homogeneity of the FA CNDs distribution over sample thickness, the MFs with dopants were scanned at various vertical positions, with a step of 2 μm (Figure 5c). We noticed that the TPEF signal is stronger in the central part of the lipid tube. The change in the width of the straight fragments of the MFs along the *z*-axis was also observed, revealing the cylindrical shape of the lamellar mesophase. The cross section of the longitudinal part of the MF is widest at its mid-height, while the diameter of the structure decreases toward both its upper and lower edges. On the other hand, the oval-ended fragments seem to be collapsed in one direction (Figure 5d). These observations are consistent with the confocal images of the sample excited using the one-photon (instead of the two-photon) scheme (Figure 4g).

The MFs are composed of DLPC, containing a fully saturated aliphatic chain and a zwitterionic headgroup with a positively charged choline residue and a negatively charged phosphate group. Considering that FA CNDs are rich in polar groups, we assign interactions between them and the hydrophilic part of DLPC to electrostatic forces as well as hydrogen bonding. In the presence of negatively charged FA CNDs, the tilt angle of the DLPC headgroup can be altered to facilitate the electrostatic attraction forces between the dopants and the quaternary ammonium cation.^99,100^ Furthermore, the oxygen-containing phosphate groups with lone-pair electrons appear as acceptors (bases) in the formation of hydrogen bonds with the protic moieties of FA CNDs (e.g., amino or hydroxyl groups).^101^

The fluorescence microscopic studies indicated that FA CNDs are present within the whole volume of MFs (with a high content of CNDs in the water core and lower contribution in the multilayered walls). Combining these results and potential interactions of hydrophilic negatively charged dopants with the phosphatidylcholine headgroups, we consider that FA CNDs are located in the aqueous phase of MFs (i.e., in the water core as well as aqueous layers between lipid bilayers) and can be adsorbed onto lipid bilayer–water interfaces.

## 3 Conclusions

Using a novel two-step protocol combining ultrasound-induced assembly of FA precursors, hydrothermal treatment of the as-formed complexes, and efficient purification process, we synthesized highly fluorescent nitrogen-doped CNDs. The prepared FA CNDs are quasi-spherical in shape with an average diameter of 7.8 nm. They possess a unique internal architecture composed of aromatic domains embedded in the polymeric matrix and are decorated with various polar moieties. Aqueous dispersions of the FA CNDs show strong blue fluorescence excitable under both the OPE and TPE schemes, with high FQY (∼54.1%) and long lifetimes (∼7.5 ns). The TPA spectrum of the FA CNDs covers the red and NIR I regions (the first biological window). The σ_TPA_/*M* merit factors of ∼0.31 GM·mol/g at 710 nm are comparable with those of the most efficient fluorescent nanoprobes reported in the literature (in a similar spectral range). The extensive microscopic studies revealed that lipidic mesophases can be doped with FA CNDs without perturbing the MFs’ formation. The uniform fluorescence observed along the multilamellar tube indicates the homogeneous distribution of FA CNDs in the membrane, regardless of the local lipids’ orientations. Combining the CFM and TPEFM techniques allowed us to get detailed information on the distribution of dopants within the MFs. Therefore, we showed that FA CNDs may be used as efficient fluorescent probes to study the morphology of the lamellar mesophases with a high spatial resolution. Using TPEFM with NIR excitation to image multilayered structures with FA CNDs is an attractive technique for further experiments on biological materials that can overcome the limitations of OPEF microscopy, as tissue components show strong absorption in UV. This way of imaging the MFs using CNDs may provide a new approach to studying demyelinating disorders.

## 4 Materials and Methods

### 4.1 Materials

Folic acid (FA), heavy water (D_2_O), methanol, ethanol, chloroform, sodium chloride, sodium phosphate dibasic anhydrous, sodium phosphate monobasic heptahydrate, and silica gel were purchased from Sigma-Aldrich. Sodium hydroxide and hydrochloric acid were purchased from POCH S.A. DLPC was purchased from Avanti Polar Lipids. Ultrapure Milli-Q water was provided by an ultrapure water system. All chemicals were stored in proper conditions and used without further purification processes.

### 4.2 Fabrication of FA CNDs

We developed a new synthesis method to fabricate the blue-emitting FA CNDs, following the facile hydrothermal route with FA molecules as nitrogen-rich carbon precursors. 50.0 mg of precursor powder (1 equiv, 113 μmol) was mixed with 7.5 mL of Milli-Q (pH = 7.0) and loaded into a round-bottom flask. The glass vessel was immersed in a water bath (25 °C) and then mixed under ultrasound treatment for 10 min (ultrasound power of 200 W and frequency of 40 kHz). The as-obtained yellow-colored aqueous dispersion was transferred to a Teflon-lined stainless autoclave reactor, which was then placed in an oven and heated up to 240 °C for 6 h. To stop the synthesis process, the reaction mixture was cooled down to room temperature in air. The sample was then filtered through a modified-cellulose membrane (MCE, pore size ∼220 nm) and freeze-dried overnight.

The crude products were dispersed in 0.5 mL of methanol, diluted with 1.5 mL of chloroform, and purified with silica column chromatography (SCC). A chloroform: methanol mixture [with volume ratio (v/v) = 3:1] and silica gel (pore size 60 Å, 230–400 mesh particle size) were used as the eluent and the stationary phase, respectively. The progress in the purification process was monitored by thin-layer chromatography (TLC) under UV-light illumination (λ = 254 and 365 nm) and UV–Vis absorption and OPEF spectroscopy. The as-obtained FA CNDs mixture was dried using a rotary evaporator system. To obtain pure FA CNDs, the collected fraction of FA CNDs was dispersed gradually in the methanol–chloroform mixture (v/v = 3:1) and purified with the SCC experiment as described above. The target fraction was dispersed in methanol and dried. The fraction of FA CNDs was dispersed in ethanol, filtered through the polyethersulfone membrane filters (PES, pore size ∼220 nm), and dried. The final product was dispersed in Milli-Q water, filtered with the MCE filters to discard aggregates, and freeze-dried. The resulting dark yellow powder of FA CNDs was stored at 4 °C for further investigations.

To optimize the strategy of FA CNDs fabrication under given thermodynamic conditions, the synthesis processes were run for varying FA concentrations in aqueous mixtures: 25 mg (56.6 μmol), 50 mg (113 μmol), 100 mg (227 μmol), 150 mg (340 μmol), and 200 mg (453 μmol). Each reaction mixture was purified, following the above-described protocol; then its extinction and fluorescence spectra were recorded and analyzed. Once the presence of the pure FA CNDs was confirmed, the reaction yields were estimated (with respect to the mass of the FA CNDs in a powder form) and normalized.

### 4.3 Structural Characterization of the FA CNDs

The structures of the FA carbon precursor and the FA CNDs were characterized by Fourier-transform infrared spectroscopy (FTIR), Fourier-transform Raman spectroscopy (FTRaman), nuclear magnetic resonance spectroscopy (NMR), and X-ray photoelectron spectroscopy (XPS). The elemental composition was determined by energy-dispersive X-ray spectroscopy (EDX) combined with scanning transmission electron microscopy (STEM). The morphology of the FA CNDs was examined with high-resolution transmission electron microscopy (HR-TEM).

The measurements of FTIR spectra of solid samples were performed in the mid-infrared (MIR; 4000–400 cm^–1^) and far-infrared (FIR; 400–50 cm^–1^) regions in the attenuated total-reflectance mode (ATR-FTIR). The MIR ATR-FTIR spectra were recorded on an IR TENSOR 27 spectrometer (Bruker), equipped with a diamond ATR crystal, a KBr beam splitter, a pyroelectric DTGS detector, and a black body source. The FIR ATR-FTIR signals were collected under vacuum conditions (2 mbar) on an IR IFS 66v/S spectrometer (Bruker), equipped with a Mylar beam splitter and silicon carbide (SiC) as an infrared (IR) source. The spectral resolutions were set to be 4 cm^–1^ in each case.

Raman signals of the FA and the FA CNDs in the solid state were acquired in the range 100–4500 cm^–1^ on an RFS100 spectrometer (Bruker) in the macro configuration, using a nitrogen-cooled Ge detector. To minimize the luminescence background, a continuous-wave Nd-YAG laser (1064 nm) was used as an excitation source. To verify the reliability of the as-obtained Raman features and samples’ stability, Raman spectroscopy scans were accumulated several times at different laser positions in each sample.

The ^1^H NMR, ^13^C NMR, and heteronuclear single quantum coherence (HSQC) NMR signals of FA CNDs in the D_2_O suspensions were measured on a Bruker Avance 600 MHz spectrometer. The NMR signals were then analyzed with the MestReNova software.

TEM samples were prepared by deposing a 10 μL drop of 5 μg/mL suspension of FA CNDs on a standard carbon film on a copper grid (300 mesh) and dried in air. The imaging of the as-prepared individual FA CNDs was performed on an HR-TEM microscope setup (JEOL 2200FS), equipped with the field emission gun (FEG) working at an accelerating voltage of 200 kV. To determine precisely the size distribution of FA CNDs, 90 nanoobjects were treated with the ImageJ software.

EDX signals of the FA CNDs were accumulated at different positions by a silicon drift EDX detector (SDD Oxford Instrument XMaxN 100 TL) combined with the STEM microscope setup (JEOL 2200FS). The representative XPS spectrum was recorded on an ultrahigh-vacuum PREVAC 426 system with a pass energy of 200 eV, equipped with a Mg/Al source (PREVAC) providing Al Kα X-rays (1486.7 eV, 150 W), an EW3000 hemispherical analyzer (VG Scienta), and an XPS EW3000 spectrometer (VG Scienta). The EDX and XPS experiments were carried out on samples that had not been ion cleaned prior to the measurements.

The zeta potential assay was performed on the multifunctional Zetasizer instrument (Nano Series, Malvern Instruments, UK), operating in the zeta potential mode. Samples were loaded into the polystyrene folded-capillary cell and incubated for 120 s before experiments. Each measurement (averaged from 10 consistent scans) was repeated six times. The raw data were examined with the 6.10 Malvern software.

The pH values of CND dispersions were monitored with a Mettler Toledo instrument (SevenCompact Series) at room temperature. To adjust a proper pH, samples were titrated with a fresh solution of 1 M NaOH or 1 M HCl, keeping the FA CNDs concentration constant.

All structural characterizations were performed at room temperature.

### 4.4 Computational Details: Density Functional Theory Calculations

The vibrational frequency analysis of precursor molecules was performed with the density functional theory. The calculations were performed with Gaussian 16, Rev. C03^102^ with the M06-L/def2-TZVP^103,104^ level of theory adopted for all atoms and the Mulliken atomic charges. The Grimme empirical correction (D3) was applied.^105^ The frequencies were scaled using an empirical scaling factor of 0.951.^106^ All input and output files are provided as a part of Supporting Information. The stationary points in the potential energy surface were confirmed not to have any imaginary frequencies. All structures were modeled in the closed-shell singlet state.

### 4.5 Linear Optical Properties of FA CNDs

The UV–Vis extinction spectra of FA molecules (C = 3.62 μM), their aggregates’ mixtures, and the FA CNDs were measured with a Jasco V-730 spectrophotometer in the 200–700 nm wavelength range. The OPE and OPEF spectra were acquired on a FluoroMax-4 spectrofluorometer (Horiba Jobin Yvon) for 3.0 nm excitation/emission slits. The one-photon excitation–emission contour maps were collected in a wide wavelength range (excitation: 200–400 nm; emission: 300–650 nm) with a spectral resolution of 1 nm. To estimate the fluorescence quantum yields (FQYs), excitation and emission spectra of the FA CNDs dispersion and blank sample were measured; a custom-built experimental setup consisted of an integrating sphere equipped with a high-sensitivity CCD array spectrometer (QE, Ocean Optics) and a BDL-375-SMN Picosecond Diode Laser (377 nm). The OPEF decays of FA CNDs were recorded with the TCSPC technique. The TCSPC detection system was composed of an Acton SpectraPro SP-2300 monochromator (Princeton Instruments), a high-speed hybrid detector HPM-100-50 (Becker&Hickl GmbH) controlled with a DCC-100 card, and a 377 nm picosecond laser diode.

### 4.6 Non-linear Optical Properties

TPEF spectra were recorded on a Shamrock 303i spectrometer (Andor) equipped with a sensitive iDus camera (Andor). A femtosecond mode-locked Ti:sapphire chameleon laser (Coherent Inc.) with a repetition rate of 80 MHz and the pulse duration of 100 fs was used as the multiphoton excitation source, operating from 690 to 1000 nm. The laser beam power was monitored with a PM100D Handheld Optical Power and Energy Meter (Thorlabs). The excitation laser beam was isolated from its reflections with a high-performance long-pass filter (FELH650, cut-on wavelength 650 nm, Thorlabs). The emitted signals were separated from the excitation light with a short-pass filter (FESH650, cut-off wavelength 650 nm, Thorlabs). To minimize inner filter and re-absorption effects, the fluorescence was measured for diluted samples, whose concentrations corresponded to absorbance values below 0.1 in the whole absorption and emission spectra ranges.

All linear and non-linear optical studies were performed for FA CNDs in aqueous dispersions (with Milli-Q water as the solvent).

### 4.7 Photostability Assays

The photostability of FA CNDs was studied by monitoring the OPEF spectra (λ_exc._ = 350 nm) of their aqueous dispersions at varying irradiation times (up to 60 min). To illuminate the samples, both the UV lamp (λ_exc._ = 365 nm) and the femtosecond laser beam (λ_exc._ = 740 nm) were used.

### 4.8 Preparation of Myelin Figures

The DLPC powder was dissolved in chloroform and vortexed. The final concentration of the lipid stock solution was 20 mg/mL. A microliter droplet of the DLPC solution was deposited on a clean microscope glass and kept overnight for residual solvent removal. The coverslip was then placed over the dried plaque of lipids. The sample thickness was set by 30 μm spacers. The as-prepared cell was filled with 1 mg/mL dispersion of the FA CNDs, and its edges were sealed with epoxy to prevent water evaporation.

### 4.9 Polarized Light Microscopy

Polarized light imaging was carried out on an Olympus BX60 optical microscope equipped with a full-wavelength retardation plate (Olympus U-TP530 Wave Plate). The micrographs were acquired with 10×/0.30 NA and 20×/0.50 NA objectives. The fluorescence images were taken by the same microscope using a 100 W mercury lamp as the light source. The dimensions of the MFs were measured from a sequence of polarized light micrographs using ImageJ.

### 4.10 Confocal Fluorescence Microscopy

The fluorescence images were taken from the confocal microscopy experiments, using a Leica TCS SP8 microscope with a 40×/0.85 NA dry objective. All samples were excited with a 405 nm laser. The fluorescence signals were collected in the emission range of 410–480 nm. The Leica Las X Software was used to perform scans recorded in three dimensions.

### 4.11 Two-Photon Excited Fluorescence Microscopy

TPEF images of the MFs were taken on a Nikon Ellipse Ti-U inverted microscope, equipped with a femtosecond laser used for the characterizations of the non-linear optical (NLO) properties of free FA CNDs. The sample was placed on the *XYZ* nanopositioning stage (Piezosystem Jena) and installed on the microscope. The 100×/1.4 NA oil immersion objective was used for the excitation beam and the fluorescence signal. The samples were excited with a 740 nm femtosecond laser beam, and the emission was collected in the 400–700 nm range. The fluorescence signal was recorded with avalanche photodiodes (IDQ id100) placed behind the filter (FF01-720/SP-25) and a dichroic beam-splitter (FF670-SDi01).

All spectroscopic and microscopic characterizations were performed at room temperature.

## Data Availability

The data that supports the findings of this study are available from the corresponding authors upon reasonable request.

## Supporting Information Available

Detailed description of spectroscopic characterizations and spectroscopic and microscopic data (PDF)

## Abbreviations

MFs: myelin figures
PLM: polarized light microscopy
CFM: confocal fluorescence microscopy
TPEFM: two-photon excited fluorescence microscopy
TPEF: two-photon excited fluorescence
TPA: two-photon absorption
NIR: near-infrared
CDs: carbon dots
FA CNDs: folic acid-derived carbon nanodots
OPE: one-photon excitation
TPE: two-photon excitation
FA: folic acid
DLPC: 1,2-dilauroyl-*sn*-glycero-3-phosphocholine
MCE: modified-cellulose membrane
SCC: silica column chromatography
TLC: thin-layer chromatography
OPEF: one-photon excited fluorescence
UV–Vis: ultraviolet–visible
PES: polyethersulfone
FTIR: Fourier-transform infrared spectroscopy
NMR: nuclear magnetic resonance
EDX: energy-dispersive X-ray
STEM: scanning transmission electron microscopy
HRTEM: high-resolution transmission electron microscopy
IR: infrared
MIR: mid-infrared
FIR: far-infrared
ATR: attenuated total-reflectance mode
HSQC: heteronuclear single quantum coherence
FEG: field emission gun
XPS: X-ray photoelectron spectroscopy
DFT: density functional theory
FQY: fluorescence quantum yield
TCSPC: time-correlated single-photon counting
NLO: non-linear optics
PT: pterin
GA: glutamic acid
PABA: *p*-aminobenzoic acid;
