# Supplementary material for: Strongly Emitting Folic Acid-Derived Carbon Nanodots for One- and Two-Photon Imaging of Lyotropic Myelin Figures
Source: ACS Appl Mater Interfaces. 2023 Jun 27;15(27):32717–31. doi: 10.1021/acsami.3c05656 (PMC10347123; doi:10.1021/acsami.3c05656)
Supplement: Supplementary file 1 — am3c05656_si_001.pdf [file am3c05656_si_001.pdf]

Electronic Supplementary Information:

Strongly emitting folic acid-derived carbon nanodots for one- and two-photon imaging of lyotropic myelin figures

Dominika Benkowska-Biernacka[1, #], Sebastian G. Mucha[2, #], Lucyna Firlej[2, 3], Filip Formalik[4,5], Jean-Louis Bantignies[2], Eric Anglaret[2], Marek Samoć[1], and Katarzyna Matczyszyn[1, *]

[1]Institute of Advanced Materials, Faculty of Chemistry, Wroclaw University of Science and Technology, 50-370 Wroclaw, Poland,

[2]Laboratoire Charles Coulomb, UMR5221, Université de Montpellier (CNRS), Montpellier, France,

[3]Department of Physics and Astronomy, University of Missouri, Columbia, Missouri 65211, United States,

[4]Department of Chemical and Biological Engineering, Northwestern University, Evanston, Illinois 60208, United States,

[5]Department of Micro, Nano, and Bioprocess Engineering, Faculty of Chemistry, Wroclaw University of Science and Technology, 50-370 Wroclaw, Poland;

# these authors contribute equally to this work,

** corresponding author;*

Table of contents:

Elaboration of synthesis protocol of FA CNDs 3
High-resolution transmission electron microscopy imaging 4
Molecular structure of folic acid molecule 6
Fourier-transform infrared spectra of FA molecules 7
Infrared spectra of FA CNDs 12
Nuclear magnetic resonance spectra of FA CNDs 13
Raman spectra of FA molecules and FA CNDs 15
Energy-dispersive X-ray spectra of FA CNDs 17
X-ray photoelectron spectra of FA CNDs 18
Zeta potential assays 19
Linear optical properties of FA precursors 21
Linear optical properties of FA-derived materials 22
pH effect on fluorescence of FA CNDs 23
Absolute fluorescence quantum yield of FA CNDs 24
Fluorescence decays of FA CNDs 25
Two-photon absorption cross-sections 26
Two-photon absorption activity of other absorbers in the first biological window 27
Two-photon brightness values of fluorophores emitting in the short-wavelength region 28
Photostability assays 28
Polarized light and fluorescence imaging of MFs 29
Experimental setup for two-photon microscopy 32
Two photon-excited fluorescence imaging of MFs 33
References: 34

Elaboration of synthesis protocol of FA CNDs

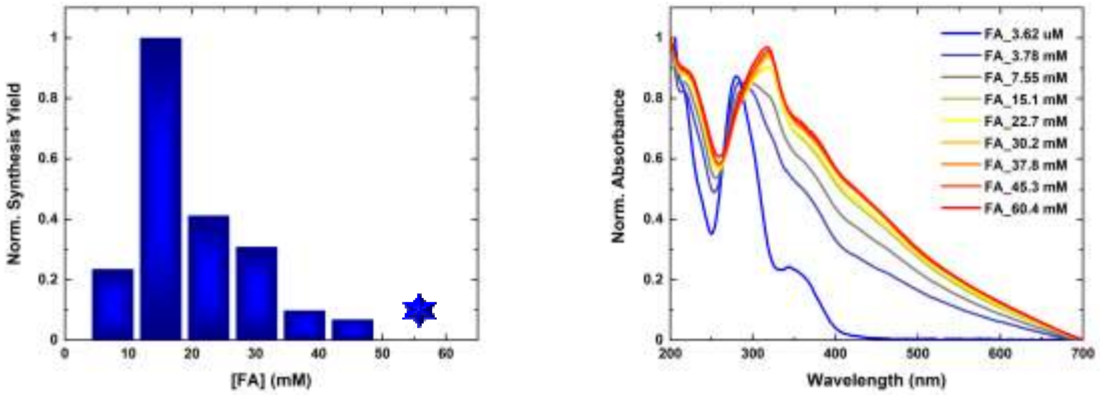

Figure S1. The evolution of normalized synthesis yields of the FA CNDs as a function of the FA molecules' amount (left). Note that the fraction of the FA CNDs was not identified for 60.4 mM, which is indicated by a blue star. The comparative evolution of extinction spectra of precursor mixture for different concentrations (right).

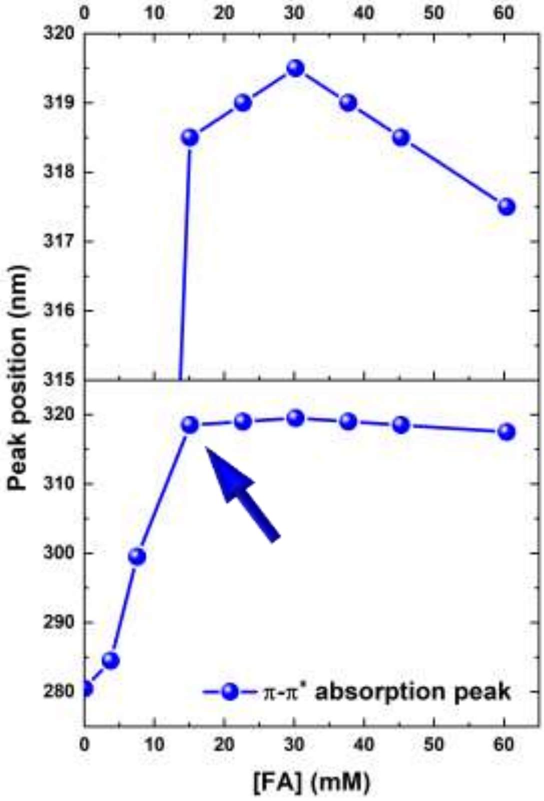

Figure S2. The correlation between peak position of the π-π* absorption band of the FA dispersions and the FA solution (first point). The precursor mixture of the target FA CNDs was indicated by an arrow.

High-resolution transmission electron microscopy imaging

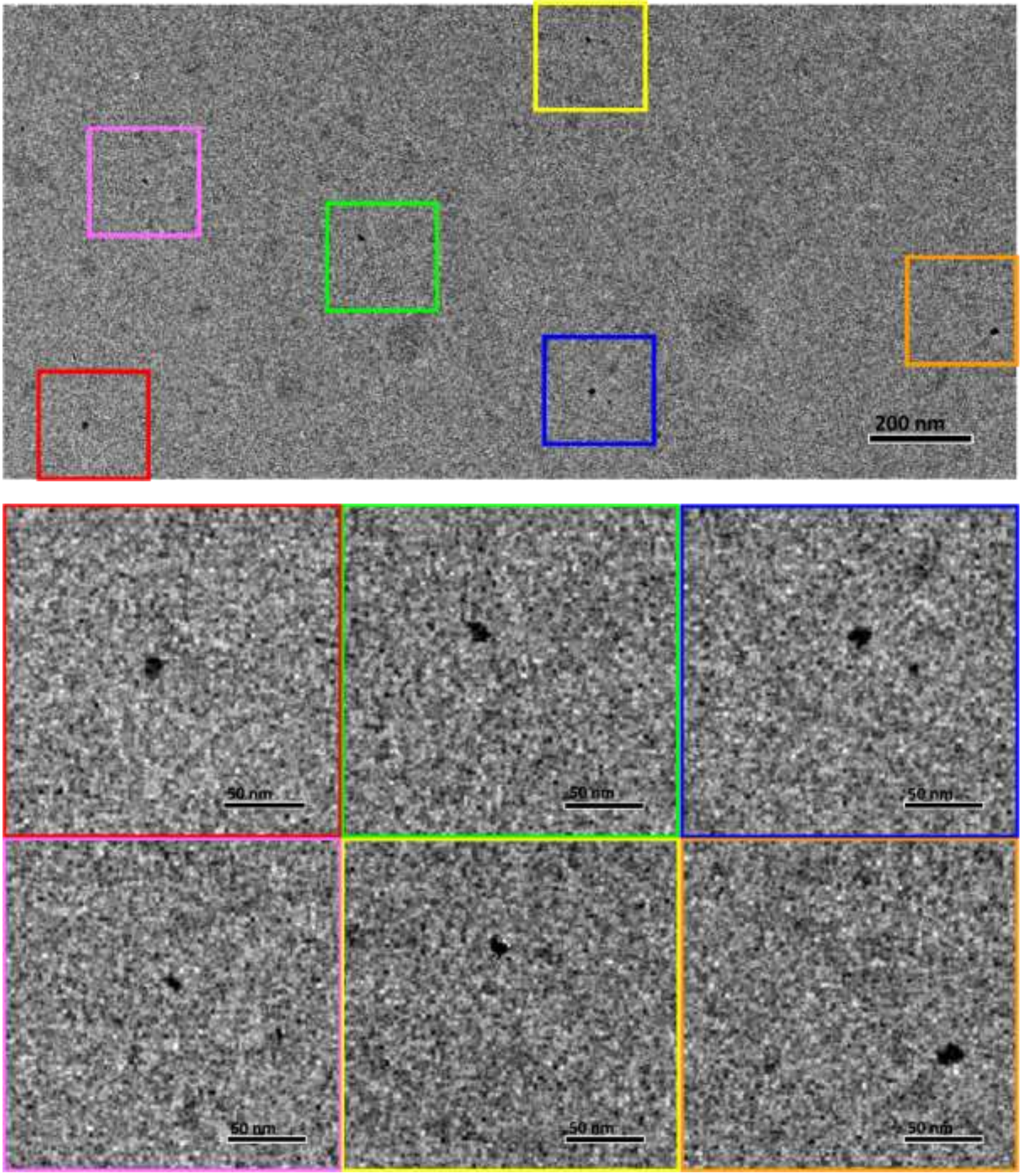

Figure S3. The HR-TEM images of the FA CNDs. The color rectangles point out representative single CNDs.

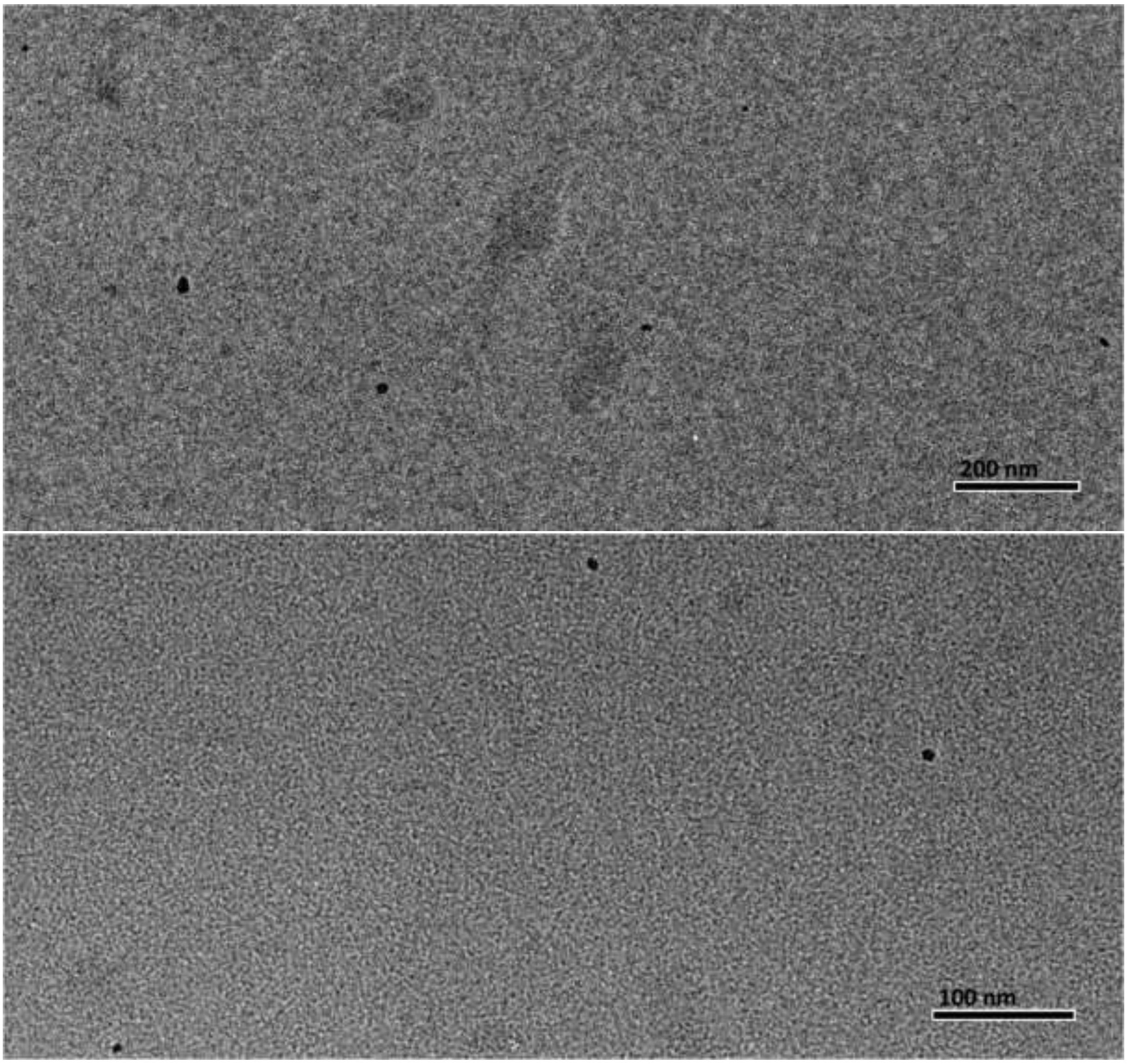

Figure S4. The HR-TEM image of single FA CNDs and an aggregation form.

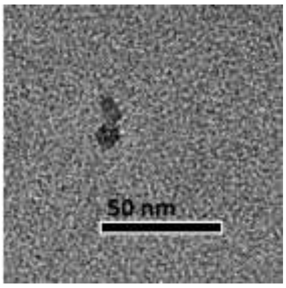

Figure S5. The HR-TEM image of dimer form of FA CNDs.

Molecular structure of folic acid molecule

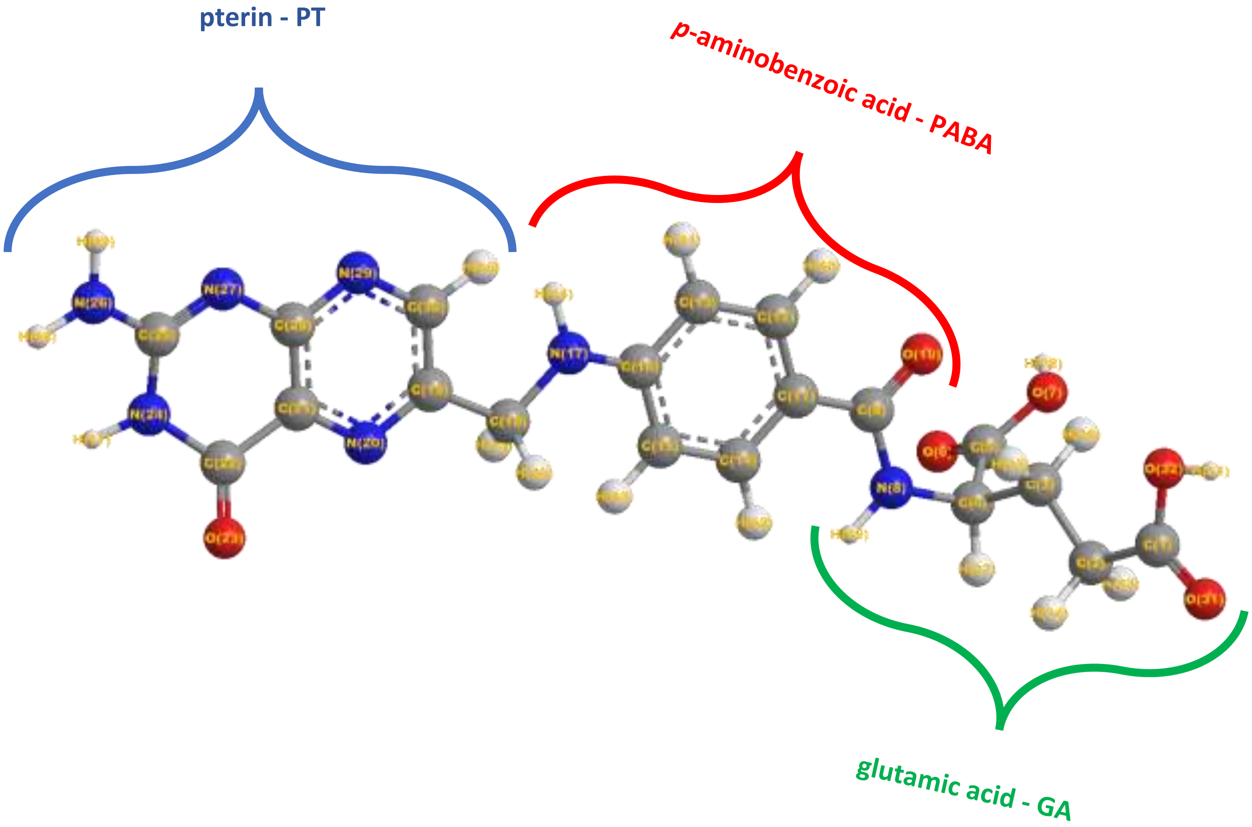

Figure S6. The molecular structure of folic acid (lactam tautomeric form) for the Raman and infrared spectroscopy analysis. Each element was indexed with a symbol (H, C, N, and O), a colour (H-white, C – grey, N – blue, and O - red), and a number (1-51). An aromaticity was indicated with dash double bonds. Three major sub-units were recognized: **pterin – PT**, ***p*-aminobenzoic acid (PABA)**, and **glutamic acid (GA)**.

Fourier-transform infrared spectra of FA molecules

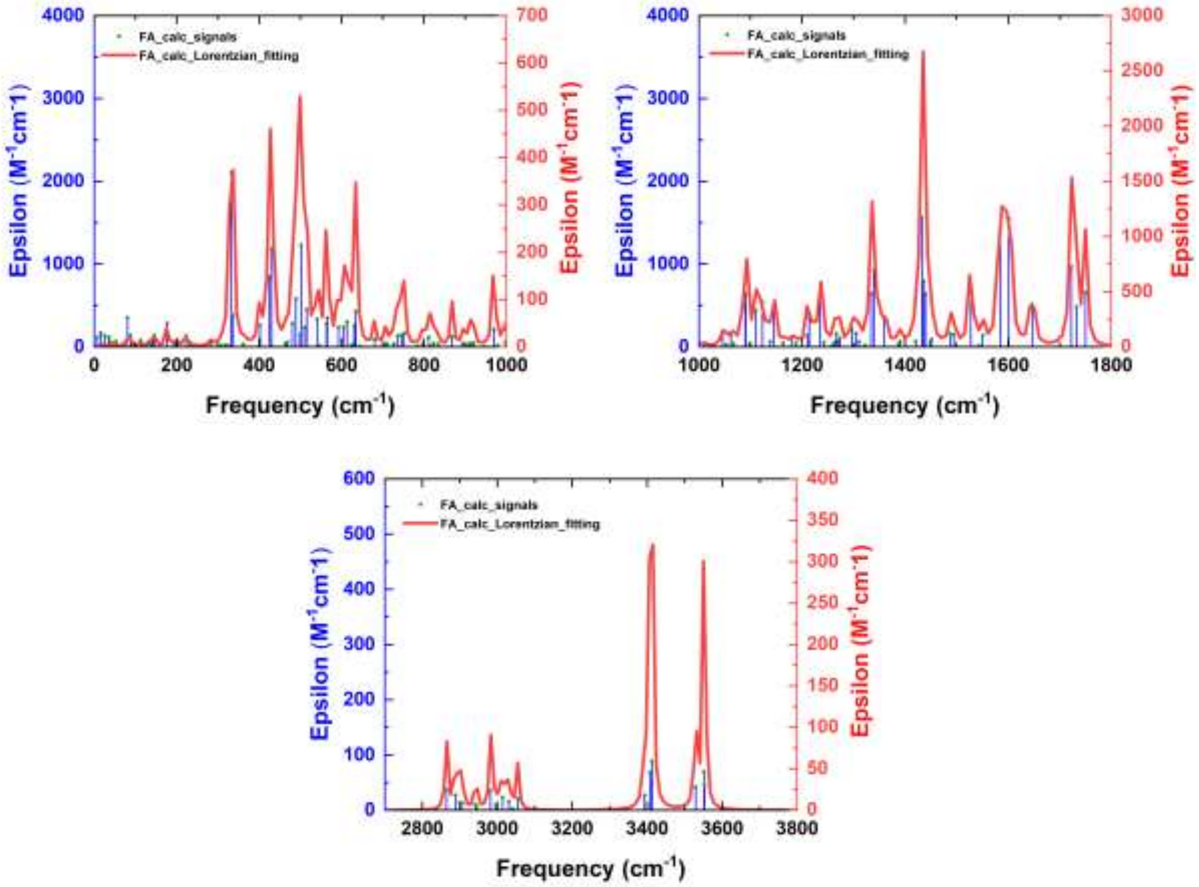

Figure S7. The calculated IR signals of a FA molecule (blue lines) fitted with the Lorentzian function (red curves). The width values of the Lorentzian peaks are 4 cm^{-1} in each case.

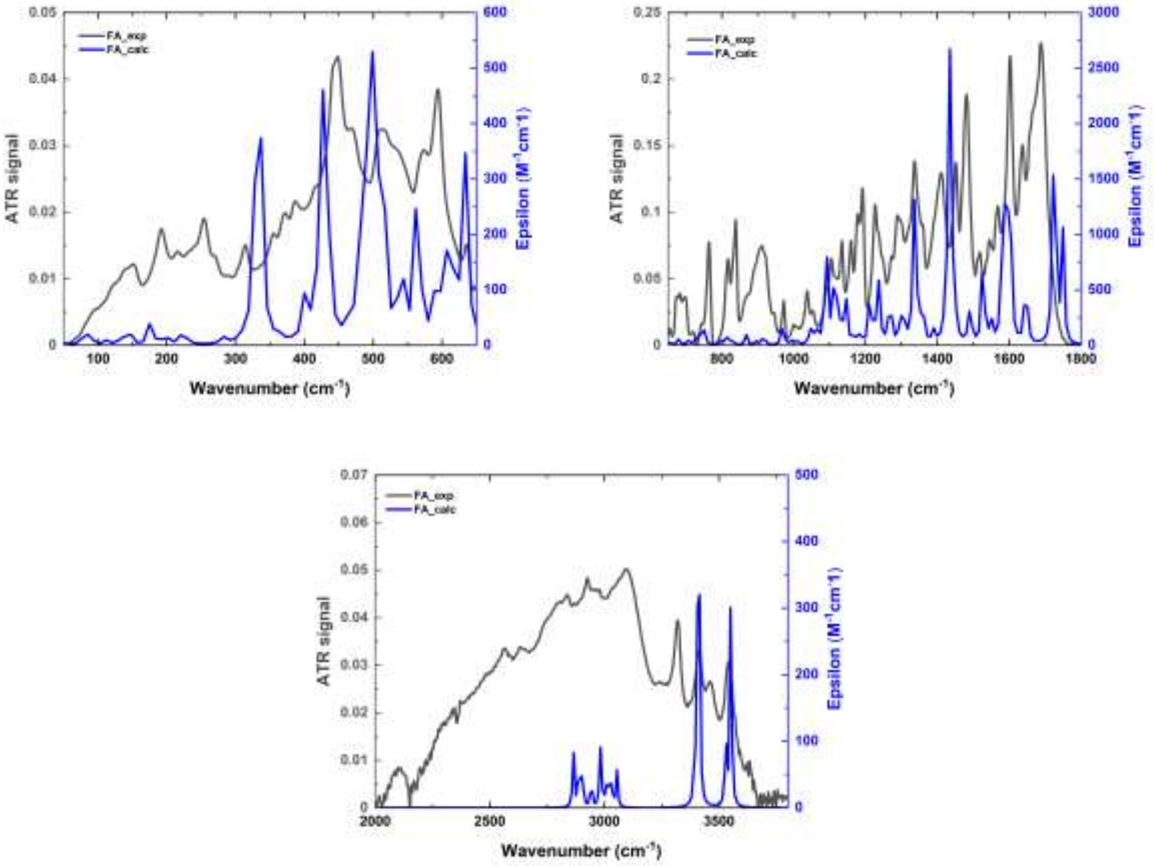

Figure S8. The comparison of experimental and theoretical IR spectra of FA molecules. Each colour of y-axis corresponds to a proper IR spectrum.

Table S1. The calculated and experimental IR signals of FA molecules. The most crucial examples were interpreted in the MIR and FIR regions (part I).

Peak position (cm^{-1})		Band assignments (major vibrations):<br>vibration mode, *chemical group*, FA sub-unit
Calculated	Experimental	
**3550-3552***	3549*	νOH *carboxylic acid*, **GA**
**3528***	3530*	νNH *amine* **PT**
**3392-3412***	3412*	νNH *amine* **PT**, **PABA**; *amide* **GA**
**-**	3100	νNH *amine*
**3014, 3030,<br>3040, 3055**	3026, 3061	νCH *aromatic*, **PABA**
**2982**	2977	νCH *aromatic*, **PT**
**2942, 2945**	2923	νCH *methylene*, **GA**
**2869, 2907**	2864, 2895	νCH *methylene*, **PABA**
**1733, 1750**	1689**	νCO, *carboxylic acid*, **GA**<br>δOH, *carboxylic acid*, **GA**<br>δNH, *amide*, **GA**
**1722**	1670**	νCO, *lactam ring*, **PT**; δNH, *lactam ring*, **PT**
**1646**	1636**	νC=C (quadrant), *aromatic*, **PABA**<br>νCO, *amide*, **PABA**; δNH, *amide*, **GA**
**1601**	1602***	νC=N, *lactam ring*, **PT**; δNH and δCO, *lactam ring*, **PT**<br>δNH, *NH_2 amine*, **PT**
**1584**		νC=C (quadrant), *aromatic*, **PABA**<br>δCH, *aromatic*, **PABA**; νCN, **PABA**
**1551**	1568	νC=N, *lactam ring*, **PT**; δNH, *lactam ring*, **PT**<br>δNH, *amine*, **PT**
**1543**	1546	δNH, *amine*, **PABA** ; δCN, *amine*, **PABA**<br>νC=C (quadrant), aromatic, **PABA**
**1528**	1518	νC=N (quadrant), *lactam and aromatic*, **PT**<br>δNH amine, δCH aromatic, δCN amine, **PT**<br>δNH, NH_2 amine, **PT**
**1488, 1494**	1481***	νC=C (quadrant), aromatic, **PABA**<br>νC=C (quadrant) aromatic, **PT**<br>νC=N (quadrant) lactam and aromatic, **PT**
**1451**	1451***	νC=N (semicircle) lactam and aromatic, **PT**<br>δNH NH_2 amine and lactam ring, **PT**<br>δCH aromatic, **PT**<br>δCH methylene, δNH amine, **PABA**
**1439**		δNH amide, δCH methylene, νCN amide, **GA**
**1432, 1436**		νC=N (semicircle) lactam and aromatic, **PT**<br>δCH aromatic, **PT**;<br>δCH methylene, δNH, amine, **PABA**; δNH, amide, **GA**;
**1390, 1421**	1410***	δCH methylene, **GA**
**1387**		νC=N (semicircle) aromatic, δNH NH_2 amine, **PABA**
**1359**	1359	ρCH methylene, νCC alkane chain, δOH carboxylic acid, **GA**<br>νCN amide, δNH amide **GA**
**1339**	1337***	
**1334**		ρCH methylene, **PABA**; νCC alkane, νC=N and νC=C aromatic, **PT**

Table S2. The calculated and experimental IR signals of FA molecules. The most crucial examples were interpreted in the MIR and FIR regions (part II).

Peak position (cm^{-1})		Band assignments (major vibrations): vibration mode, chemical group, FA sub-unit
**Calculated**	**Experimental**	
**1322**	1322***	νC=C (semicircle) aromatic, δCH aromatic, δCN amine, δCC alkane, νCO amide, ρCH methylene, **PABA**; νC=C (semicircle) aromatic, νCC alkane, δNH lactam, **PT**
**1310**	-	νCO, δCO and δOH carboxylic acid, νCN amide, ρCH methylene, νCC alkane, **GA**
**1297, 1305**	1297	νC=C (semicircle) aromatic, δCH aromatic, νCN amine, ρCH methylene, **PABA**; νC=N (semicircle) aromatic, δCH aromatic, νC=N lactam ring, δNH lactam and amine, **PT**
**1273**	1274***	νC=C (semicircle) aromatic, δCH aromatic, νCN amine, δNH **PABA**; νC=N lactam ring, δNH lactam and amine, νC=N (semicircle) aromatic, νC=C (semicircle) aromatic, δCH aromatic, νCO lactam ring, **PT**
**1270**		νCN amide, δCO amide, νCC alkane, ρCH methylene, νCO, δCO, and δOH carboxylic acid, **GA**;
**1267**		νC=C (semicircle) aromatic, δCH aromatic, νCN amine, **PABA**
**1235, 1243**	1236, 1245	νCN amide, δCO amide, νCC alkane, ρCH methylene, νCO and δOH carboxylic acid, **GA**; νC=C (semicircle) aromatic, δCH aromatic, νCC alkane, νCN amine, δNH amine, ρCH methylene, **PABA**;
**1188**	1191	νCN and δNH amide, νCO and δOH carboxylic acid, νC-C alkane, ρCH methylene **GA**; ν ring and δCH aromatic, νC-C alkane, νCN amine, δNH amine, ρCH methylene, **PABA**;
**1179**	1179	νCN and δCO lactam, δCH aromatic, νC=C and C=N aromatic, **PT**; ρCH methylene, δNH amine, νCN amine, **PABA**
**1163**	1160	νCN amide, νCC alkane, ρCH methylene, ρNH amide, **GA**; δCH aromatic **PABA**;
**1138**	1134	νCN lactam and aromatic ring, δCO lactam **PT**; ρCH methylene, **PABA**
**1110, 1123**	1105, 1118	νCN and δNH amide, νCO and δOH carboxylic, ρCH methylene, **GA**
**1053**	1052	νCN lactam and aromatic ring, δNH NH_2 amine, δCO lactam **PT**
**1010, 1046**	1010, 1039	ρOH carboxylic acid, νCC and ρCH methylene, ρNH amide, **GA**
**997**	1000	νCN lactam ring, δNH NH_2 amine and lactam ring, **PT**
**969**	972	ν ring aromatic, ρCH methylene, **PABA**; ρCH aromatic, νCN and νCC lactam ring, δC-C alkane, **PT**
**945**	944	ring deformation, ρCH aromatic, **PABA**
**920**	-	ν ring lactam and aromatic, ρCH aromatic, **PT**
**909**	910	ν ring, ρCH methylene and aromatic, **PT**
**867, 894**	867, 894	νring, νC-C alkane and carboxylic acid, ρCH methylene, δOH carboxylic acid **GA**
**834**	838	ν ring (breathing), **PABA**
**823**	817	ring deformation, **PT**; ρCH aromatic, ring deformation, **PABA**
**752**	764	ring deformation, **PABA**; CCN backbone deformation, **PABA**; ρCH methylene, CCO backbone deformation, **GA**

Table S3. The calculated and experimental IR signals of FA molecules. The most crucial examples were interpreted in the MIR and FIR regions (part III).

Peak position (cm^{-1})		Band assignments (major vibrations): vibration mode, chemical group, FA sub-unit
**Calculated**	**Experimental**	
**745**	744	ν ring, **PABA**; CCN backbone deformation, **PABA**; ρCH methylene, CCO backbone deformation, **GA**
**680**	683	ρCH aromatic, ring deformation, **PABA**; CCN backbone **PABA**
**634**	636	δOH carboxylic acid, CCC backbone, NCC backbone, **GA**
**592**	594	δCO lactam ring, δCN lactam ring, ring deformation, **PT**
**515**	516	ρNH amide, **GA**; ρCH aromatic and ring deformation, **PABA**
**510**	512	ρNH NH_2 amine, **PABA**; ring deformation, **PABA**; ν ring, **PT**
**463, 469**	468	ν ring, **PT**; ring deformation, **PABA**; skeleton vib., **GA**
**431**	439	ρNH NH_2 amine, **PABA**; skeleton vib. **PT**, **PABA**, **GA**
**424**	417	ρOH carboxylic acid, skeleton vib., **GA**; ring deformation, ρNH NH_2 amine, **PABA**
**361**	356	ring deformation, **PT**, **PABA**
**-**	255	COO^- amino acids, **GA******
**232**	235	skeleton vib, **PABA**; τ CCO backbone, **GA**
**223**	216	skeleton vibration, **PT**, **PABA**, **GA**
**194**	192	τ NCC backbone, **GA**
**146**	151	τ aromatic and lactam rings, **PT**; τ CCCO backbone, **GA**
**92**	96	τ CCO backbone, **GA**

Labelling: ν - stretching; δ – in-plane bending; ρ – out-of-plane bending, τ – torsion, vib. - vibration; **GA** - glutamic acid; **PT** - pterin; **PABA** - p-aminobenzoic acid;

* - stretching signals of OH and NH groups which are free of hydrogen bonds; other IR bands involving the hydrogen-bonding network from the experimental spectrum are not listed;

** - positions of those peaks are affected by inter- and intramolecular interactions;

*** - those peaks include other IR signals whose maxima cannot be indicated;

**** - the band assignment is based on the interpreted experimental data;

Infrared spectra of FA CNDs

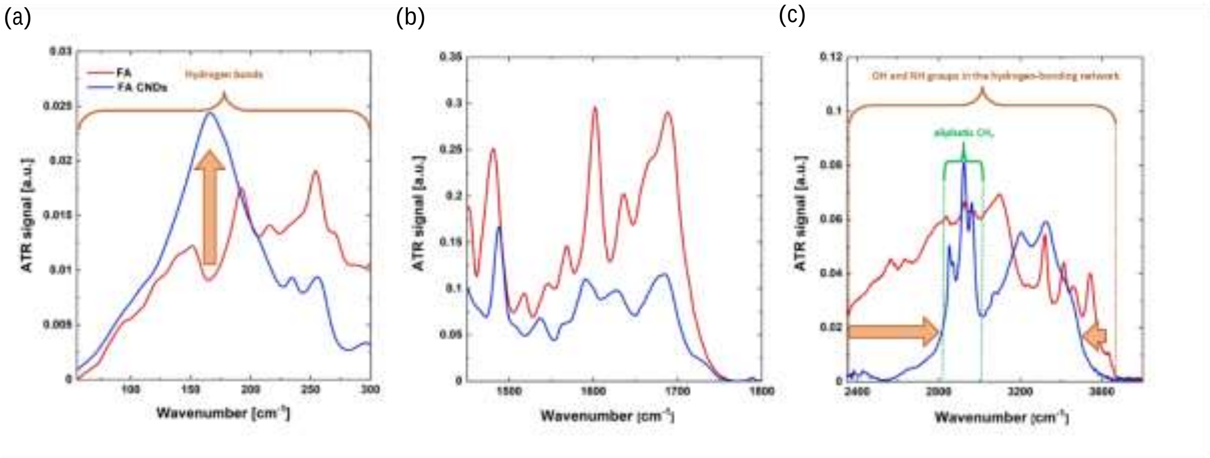

Figure S9. The ATR-FTIR spectra of the FA and the FA CNDs in the FIR and MIR regions. The evolution of the hydrogen-bonding network bands is indicated with orange arrows.

Nuclear magnetic resonance spectra of FA CNDs

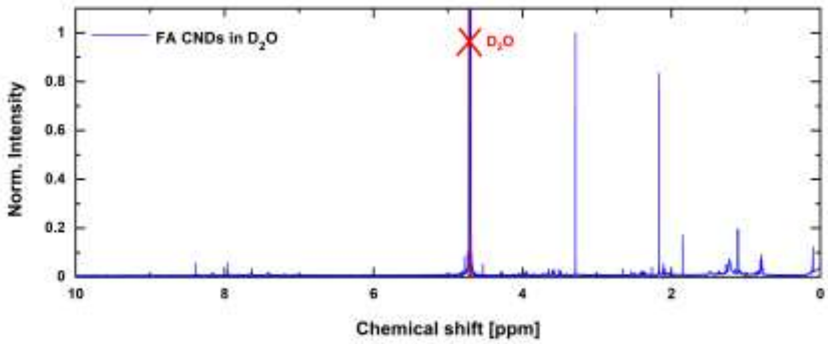

Figure S10. The ^{1}H NMR spectrum of FA CNDs in D_2O. The signal of **D_2O** is indicated. The NMR peaks of a sample were normalized with the respect to the most intense one.

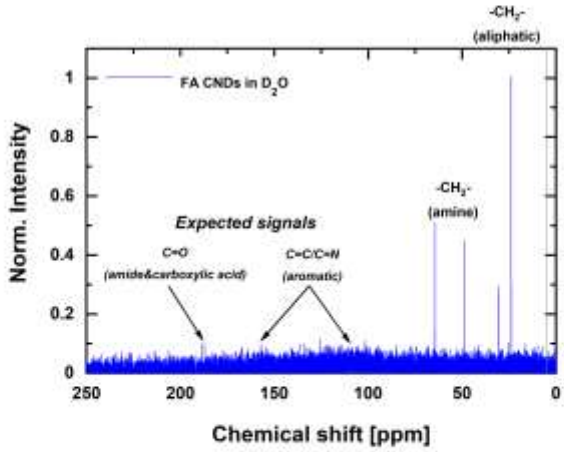

Figure S11. The ^{13}C NMR spectrum of FA CNDs in D_2O. Each NMR peak is normalized in relation to the strongest signal. The NMR signatures and the expected signals are indicated.

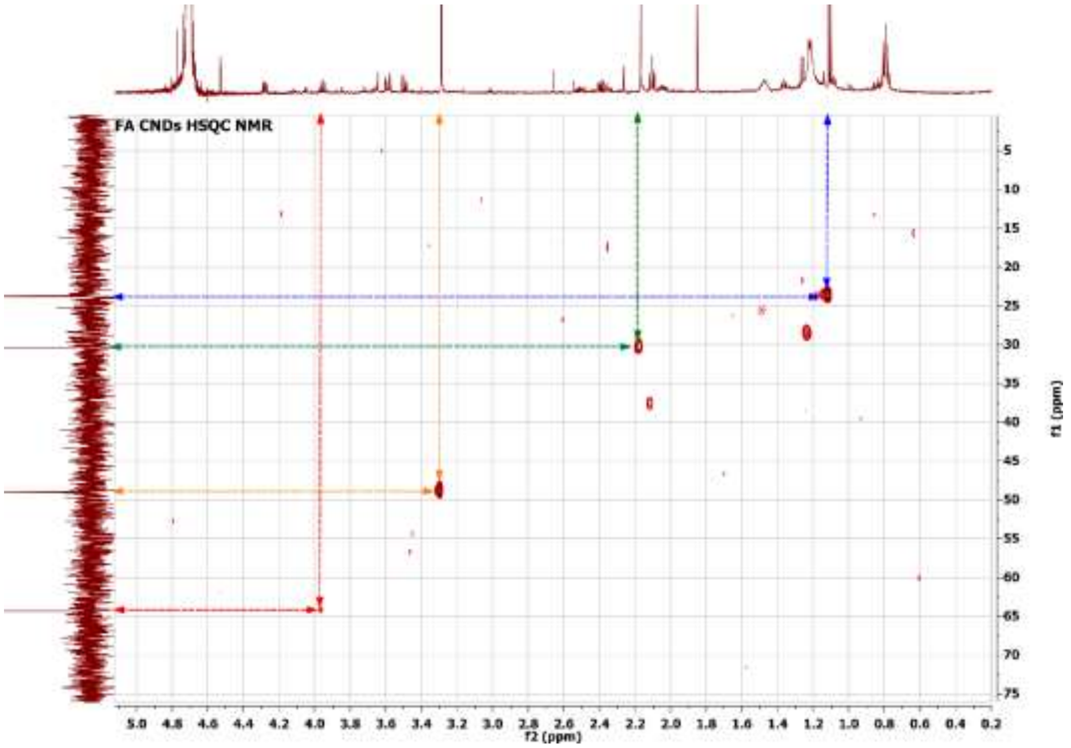

Figure S12. The HSQC NMR spectrum of FA CNDs in D_2O. The correlation between particular peaks are marked with dash arrows.

Raman spectra of FA molecules and FA CNDs

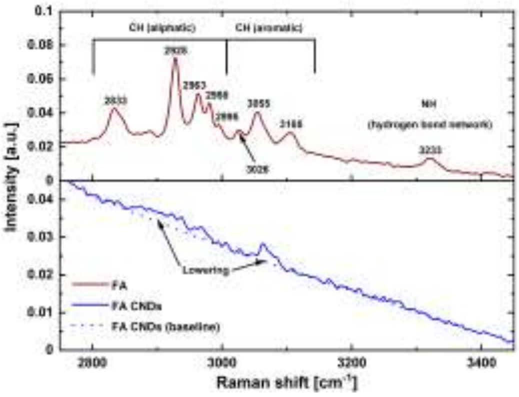

Figure S13. The Raman spectra of the FA and the FA CNDs in the high-wavenumber region. The most crucial signals of the FA are identified and compared with carbon nanodots' peaks.

Table S4. The signature of major Raman peaks of FA molecules. The Raman components are identified according to the literature.[1-3] The particular chemical groups are assigned to one of the three sub-units of a FA molecule (**PT**, **PABA**, and **GA**).

Peak position (cm^{-1})	Band assignments
3230 cm^{-1}	Hydrogen bonded **NH stretching** in secondary amides (**PABA**-**GA**);
3000-3120 cm^{-1}	**CH stretching** in aromatic rings;
2800-3000 cm^{-1}	**CH stretching** in methylene and methyl groups;
1665 cm^{-1}	**CO stretching** in secondary amides (**GA**) ;
1510-1650 cm^{-1}	**C=N stretching** in heteroatomic aromatic ring with OH rocking and NH_2 scissoring in **PT**;<br>**C=C stretching** in phenyl ring with CH bending and NH rocking in **PABA**;
1456-1488 cm^{-1}	**C=C stretching** with NH and CH bending, CH_2 scissoring in **PABA**;<br>**CH bending** in aliphatic chain in **PABA**;<br>NH rocking and CN scissoring in amide in **GA**;
1422 cm^{-1}	**C=N stretching** with CH rocking, OH rocking and CH_2 scissoring in **PT**;
1356 cm^{-1}	**C=N stretching** with CH rocking, OH rocking and CH_2 wagging in **PT**;<br>**C=C, C=N stretching** in pyrimidine ring (**PT**);
1227, 1250,<br>1291 cm^{-1}	**CH rocking** in **PABA**;<br>CH, NH, and OH rocking; CH_2 wagging in **GA**;
1179 cm^{-1}	**CH_2 twisting** with amine group;<br>**CO stretching** in carboxylic acid;

Energy-dispersive X-ray spectra of FA CNDs

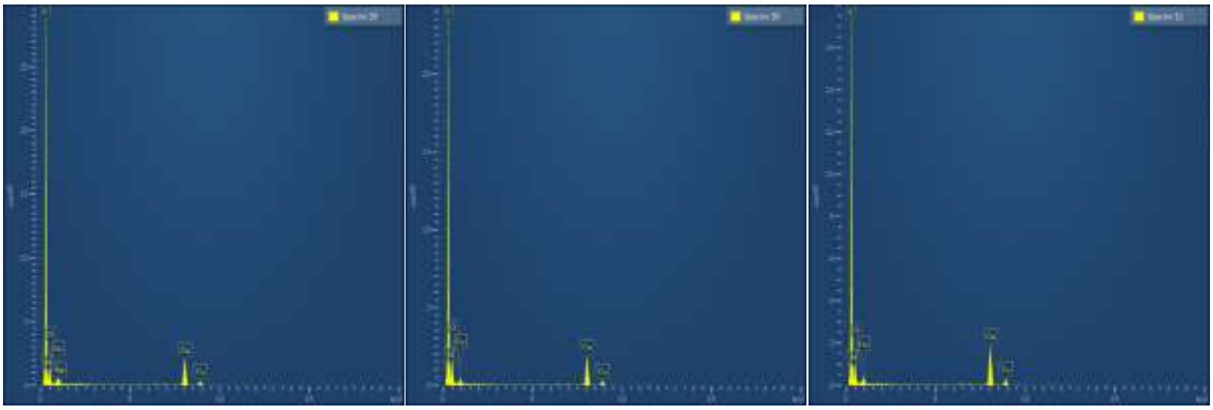

Figure S14. The representative EDX survey of the FA CNDs. Salt traces from the FA precursors (NaCl) and a copper grid component (Cu) are also present.

Table S5. The atomic contribution of heteroatoms in FA CNDs.

Heteroatom	Atomic percentage (%)					
	FA CNDs					FA ($C_{19}H_{19}N_7O_6$)
	Spectre 28	Spectre 29	Spectre 30	Spectre 31	Average value (±SD)	Theoretical value
**N**	29.92	33.32	29.06	28.53	**30.21±2.15**	**53.85**
**O**	70.08	66.68	70.94	71.47	**69.79±2.15**	**46.15**

X-ray photoelectron spectra of FA CNDs

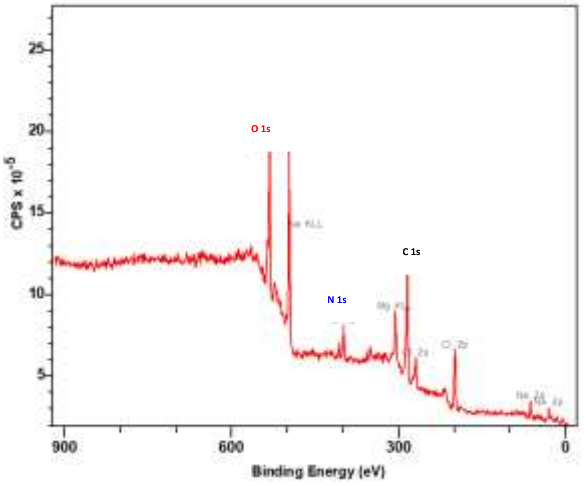

Figure S15. The XPS survey of the FA CNDs. The crucial FA CNDs' components are highlighted.

Zeta potential assays

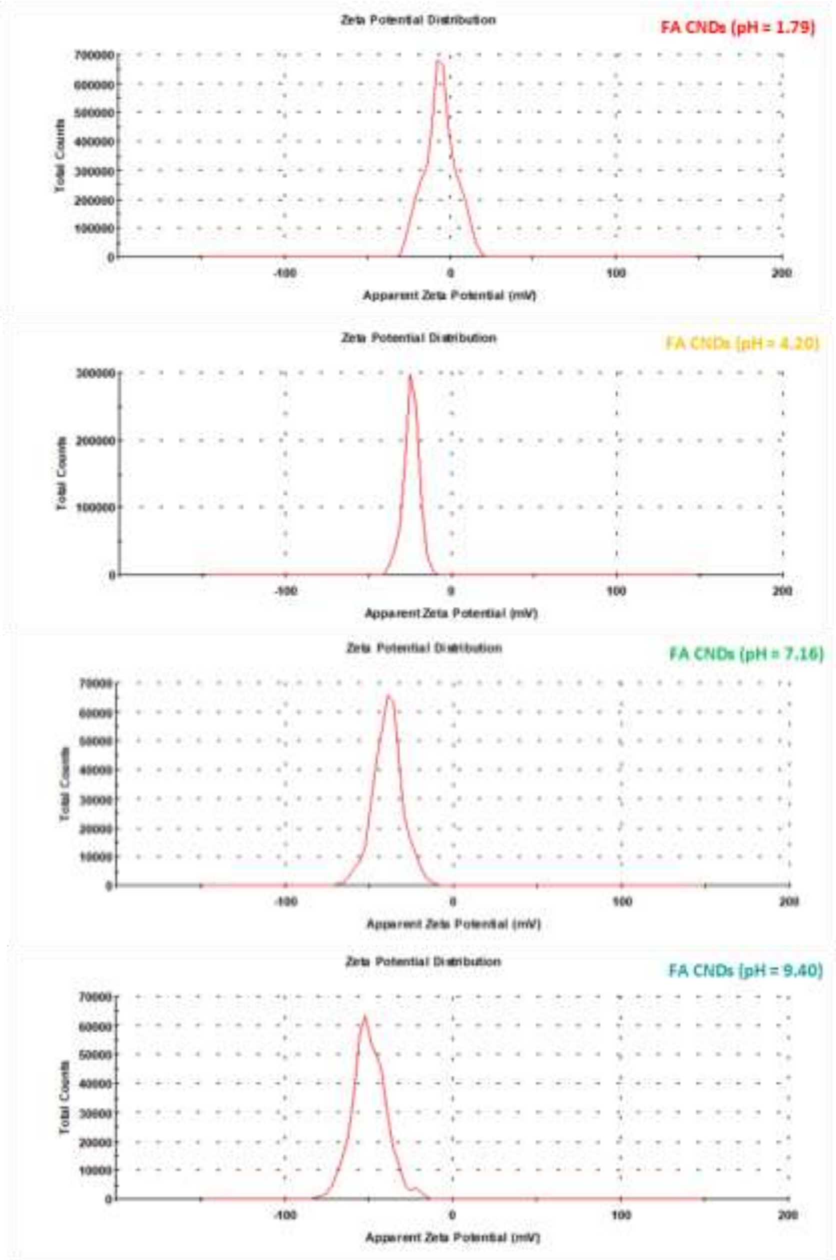

Figure S16. The Zeta potential values of FA CNDs in the aqueous media with varying pH (part I).

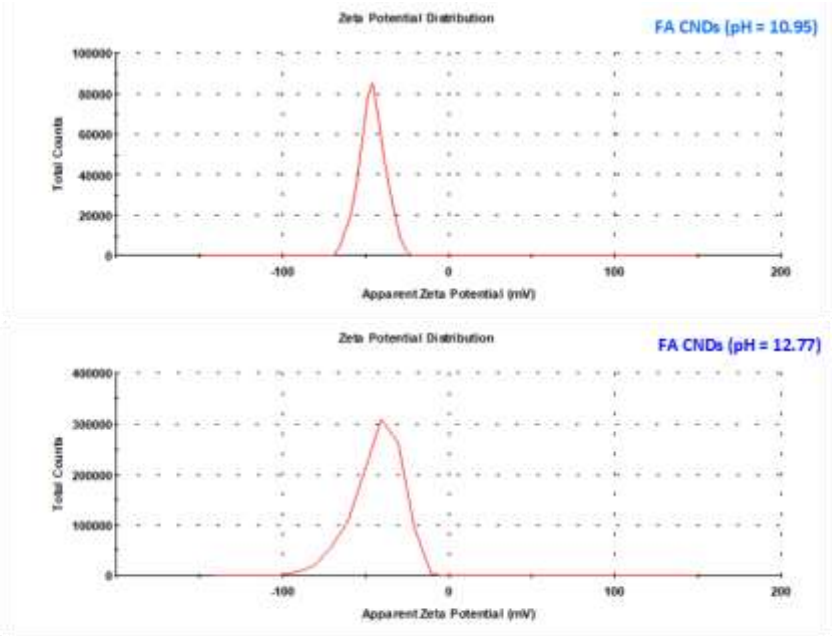

Figure S17. The Zeta potential values of FA CNDs in the aqueous media with varying pH (part II).

The evolution of the Zeta potential *vs.* the pH was fitted with the Boltzmann sigmoidal equation:

$$\zeta = \frac{\zeta_1 - \zeta_2}{1+\exp\left(\frac{pH - pH_0}{dx}\right)} + \zeta_2 \text{ (equation 1)}$$

where ζ is the varying Zeta potential (mV); ζ_1 and ζ_2 denote threshold (upper and lower) values of the Zeta potential (mV); pH is the varying parameter; pH_0 is the pH value at which the ζ is in the center between its thresholds; dx is the slope which describes a gradual change of the ζ value between ζ1 and ζ2 as a function of the pH;

Table S6. The fitting parameters from the Boltzmann sigmoidal equation:

Fitting parameter (unit)	Value
ζ_1 (mV)	-2.64
ζ_2 (mV)	-42.9
pH_0	4.50
dx	1.00

Linear optical properties of FA precursors

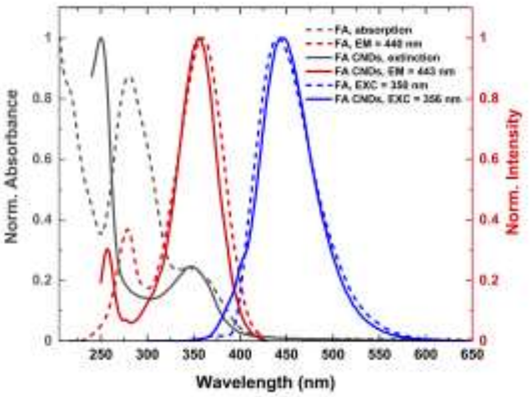

Figure S18. The normalized one-photon absorption (OPA, dark grey), one-photon excitation (OPE, red), and one-photon excited fluorescence (OPEF, blue) of FA molecules in aqueous solution (dash lines) and the FA CNDs' dispersion (solid lines). The FA molecules were dissolved to rich concentration below their solubility in water.

Linear optical properties of FA-derived materials

Table S7. The OPEF characteristics of FA molecules and FA-derived carbon nanomaterials.

Materials	OPE emission peak	FQY (%)	‹τ› (ns)	References
**FA molecules** (neutral form)	**445 nm**	0.045	2.89[a]	Tyagi *et al.*[4]
**FA molecules** (anion form)	**455 nm**	<0.5	3.5	Thomas *et al.*[5]
**FA molecules** (cation form)	**445 nm**	<0.5	7.0	Thomas *et al.*[5]
**FA CNDs**	**446 nm**	54.1	7.47	This work
**FA CDs**	**400 nm**	94.5	15.38	Liu *et al.*[6]
**FA CDs**	**440 nm**	85.0	4.86	Wang *et al.*[7]
**FA CDs**	**~450 nm**	44.4	-	Bakier *et al.*[8]
**FA CDs**	**~450 nm**	9	-	Bhunia *et al.*[9]
**FA CDs**	**~450 nm**	8	-	Sarkar *et al.*[10]
**FA CDs**	**499 nm**	27	5.84	Campos *et al.*[11]
**FA CDs**	**485**	48.2	-	Phan et al.[12]

[a] The intensity-weighted average fluorescence lifetime was calculated following lifetime components.[4]

pH effect on fluorescence of FA CNDs

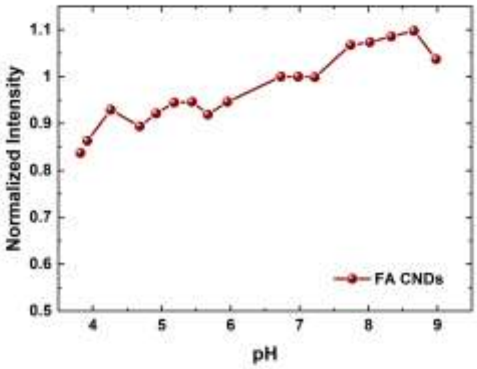

Figure S19. The evolution of the normalized OPEF response for the varying pH values.

Absolute fluorescence quantum yield of FA CNDs

The absolute fluorescence quantum yield (FQY) values of the FA CNDs were estimated, following the formula:

$$FQY = 100\% \cdot \frac{S_1 - S_0}{S_2 - S_3} \qquad \text{(equation 2)}$$

where *FQY* denotes the absolute fluorescence quantum yield (%), S_1 and S_0 are the integrated emission intensities in the presence of sample and blank sample (a.u.), S_2 and S_3 correspond to the integrated intensity in the excitation peak for a blank sample and a sample, respectively.

Fluorescence decays of FA CNDs

The fluorescence decays of the FA CNDs were fitted with a commercial software package (SPCImage, Becker&Hickl) taking into account for the collected instrumental response function (IRF) and using the bi-exponential equation, as follows:

$$I(t) = I_0 + A_1 exp\left(\frac{t-t_0}{\tau_1}\right) + A_2 exp\left(\frac{t-t_0}{\tau_2}\right) \qquad \text{(equation 3)}$$

where *I(t)* is the fluorescence intensity (a.u.), *t* represents time (ns), τ_1 and τ_2, denote lifetime components (ns), and $A_1$and A_2 are the relative decay amplitudes.

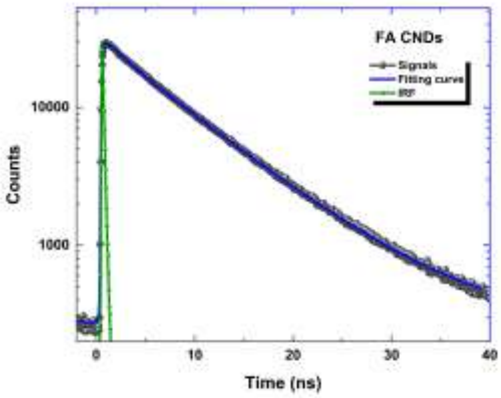

Figure S20. The fluorescence decay profile of FA CNDs (black dots) with the fitting curve (blue) and the instrumental response function (IRF; green).

As-obtained fluorescence decay parameters were averaged over six accumulations and then, involved in the estimations of the weighted average fluorescence lifetime:

$$\langle\tau\rangle = \frac{\sum A_i \tau_i^2}{\sum A_i \tau_i} \qquad \text{(equation 4)}$$

where τ_i is the lifetime *i*-component (ns), A_i denotes a properly normalized amplitude, and *‹τ›* is the calculated average lifetime (ns);

Table S8. The fluorescence lifetime characteristics of FA CNDs.

Parameter	τ_1 (ns)	A_1 (%)	τ_2 (ns)	A_2 (%)	‹τ› (ns)
Value	2.05	14.2	7.71	85.8	7.47

Two-photon absorption cross-sections

$$\frac{\sigma_{TPA,s}}{M} = \sigma_{TPA,r}\frac{I_s C_r \varphi_r n_s}{I_r C_s \varphi_s n_r} \qquad \text{(equation 5)}$$

where σ_{TPA}/M is the molar-mass scaled two-photon absorption (TPA) cross-section (GM·mol/g); σ_{TPA} denotes TPA cross-section (GM), I stands for the integrated two-photon excited fluorescence intensity (a.u.); n and φ are the refractive index of the solvent and the FQY, respectively; C is the molar concentration (reference; mol/dm^3) or the mass concentration (sample, g/dm^3). Subscripts *r* and *s* indicate reference and sample, respectively.

Two-photon absorption activity of other absorbers in the first biological window

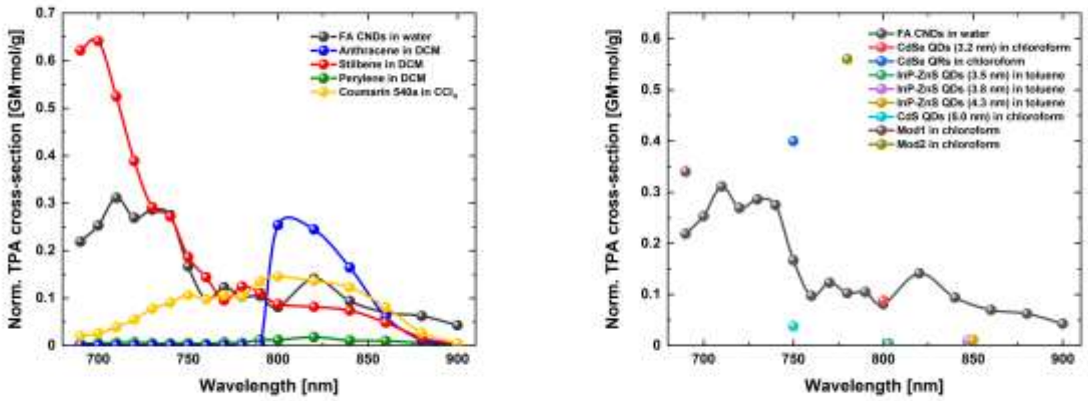

Figure S21. Comparison of normalized TPA cross-sections of organic molecules, macromolecules, and nanomaterials in the first biological window[13-18]. *Anthracene* and *Stilbene* correspond to 9-chloroanthracene and 4,4'-Bis-(diphenylamino)stilbene, respectively. QDs and QRs denote quantum dots and quantum rods, respectively. The non-linear optical parameters of organic fluorophores were estimated by dividing the TPA cross-sections by their molar mass values (in g/mol).

Two-photon brightness values of fluorophores emitting in the short-wavelength region

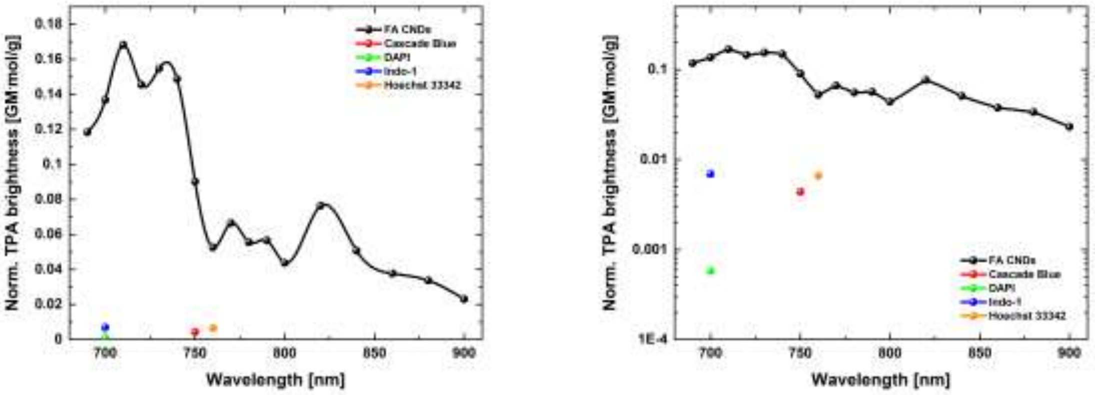

Figure S22. The TPA brightnesses presented as the molar-mass normalized values of the FA CNDs in comparison to potential blue-emitting biomarkers in aqueous solutions. The corresponding NLO parameters were recalculated from the literature data taking into account the molar mass of a given organic dye[19-21].

Photostability assays

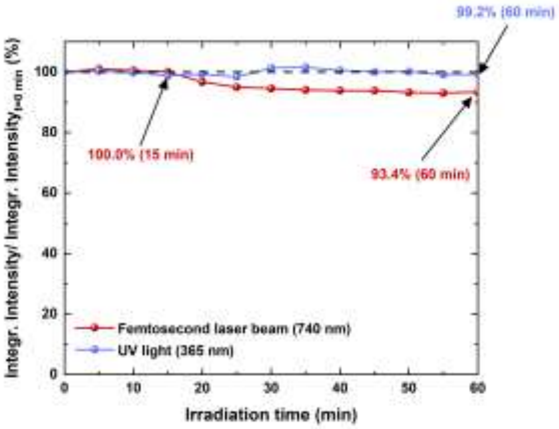

Figure S23. The evolution of the integrated fluorescence intensity ($\lambda_{exc.}$ = 350 nm) at different time intervals of continuous high-power irradiation with the UV light (365 nm; blue) and a femtosecond laser beam (740 nm; red). Each integrated intensity was normalized with respect to the intensity value of the non-illuminated dispersion. The selected normalized values are indicated with arrows.

Polarized light and fluorescence imaging of MFs

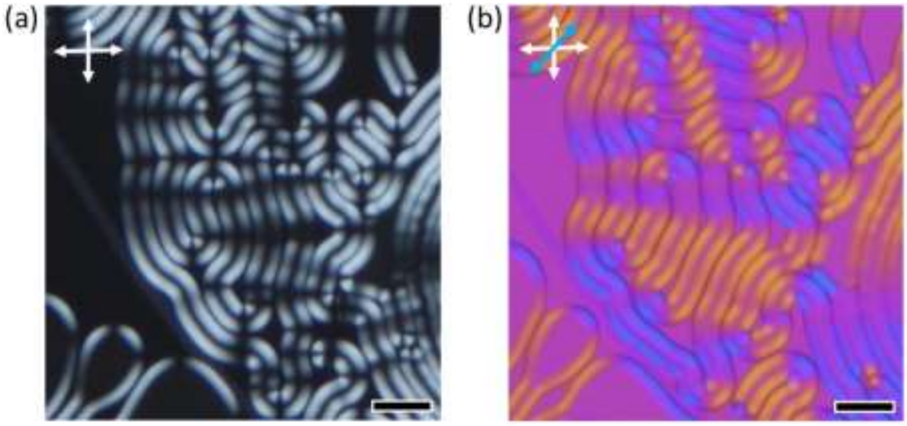

Figure S24. Representative polarized light microscopy image of undoped phosphatidylcholine-based myelin figures taken (a) without and (b) with a full-wavelength retardation plate. The arrows indicate the orientations of crossed polarizers (white double arrows) and the full-wavelength retardation plate's slow axis (blue double arrow). The scale bar is 20 µm.

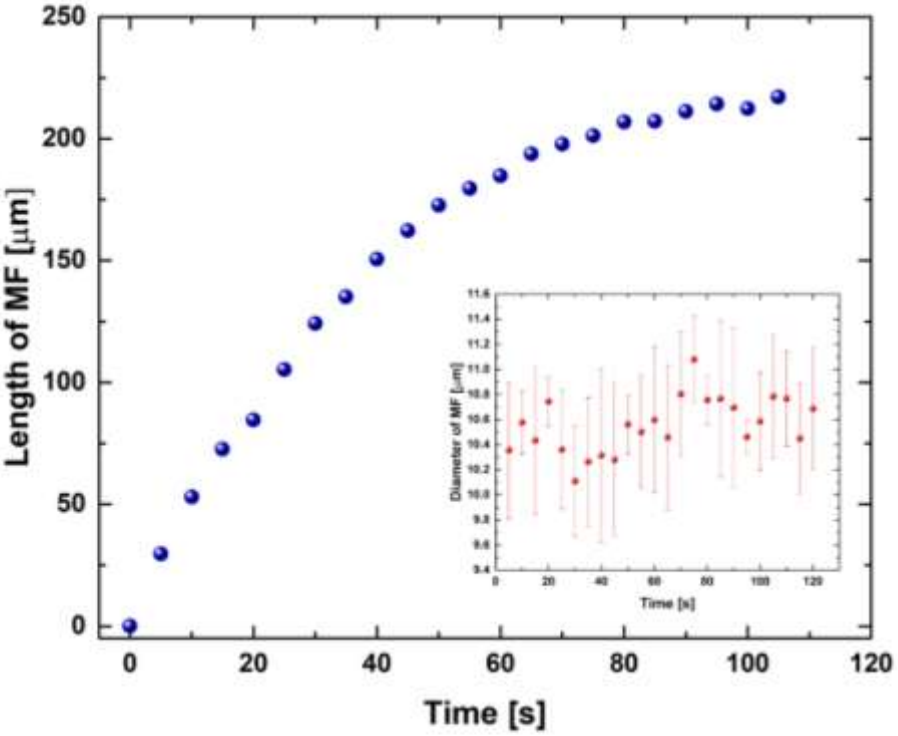

Figure S25. The time evolution of the length of the representative MF doped with FA CNDs during the growth process. The insert shows the average diameter values of the single MF measured at 5 different points along the same lipid tube versus time after sample preparation. The lengths and diameters were measured using images taken with a polarized light microscope.

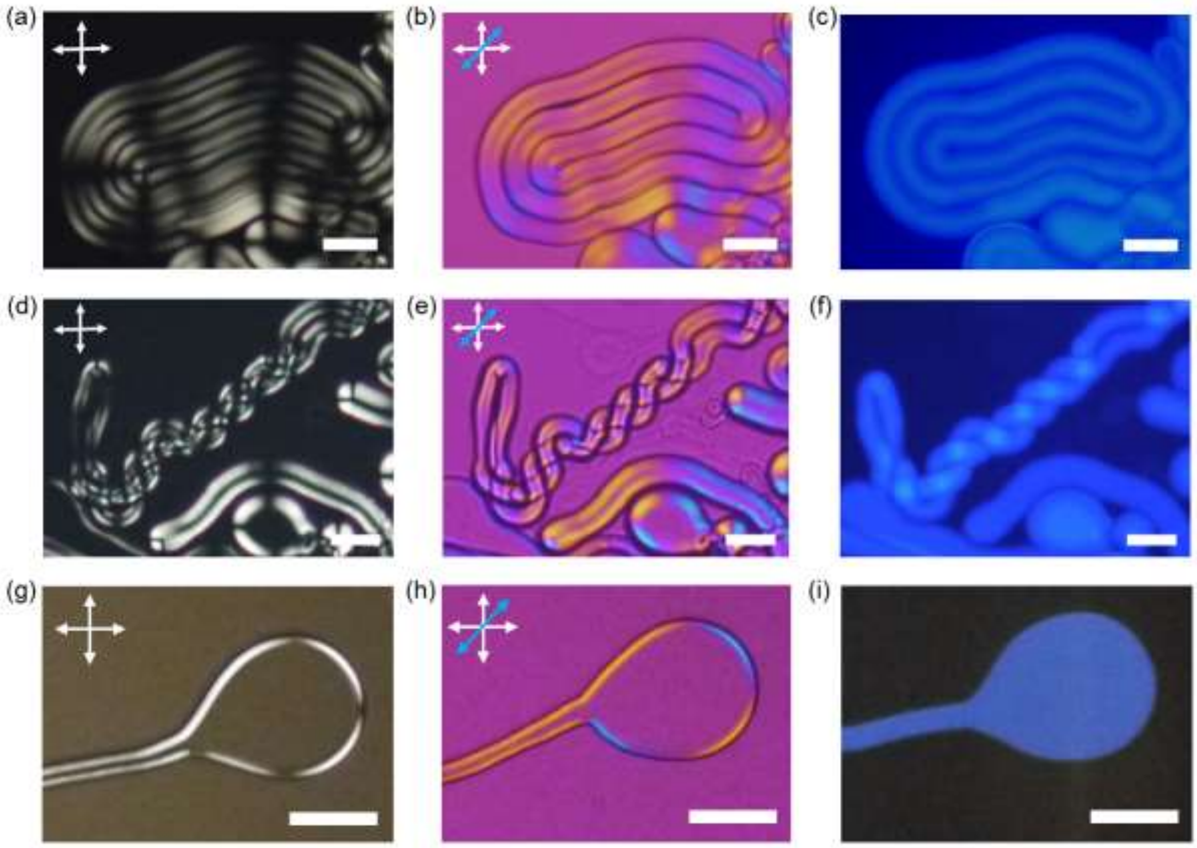

Figure S26. Micrographs of (a-c) curved, (d-f) looped and (g-i) oval-shaped MFs doped with the FA CNDs. The images taken under the polarized light microscope (a, d, g) without and (b, e, h) with the full-wavelength retardation plate. (c, f, i) The fluorescence images of the corresponding areas of the samples ($\lambda_{exc.}$ = 360 nm). The scale bars represent 10 µm in (a-c), 20 µm in (d-f) and 40 µm in (g-i).

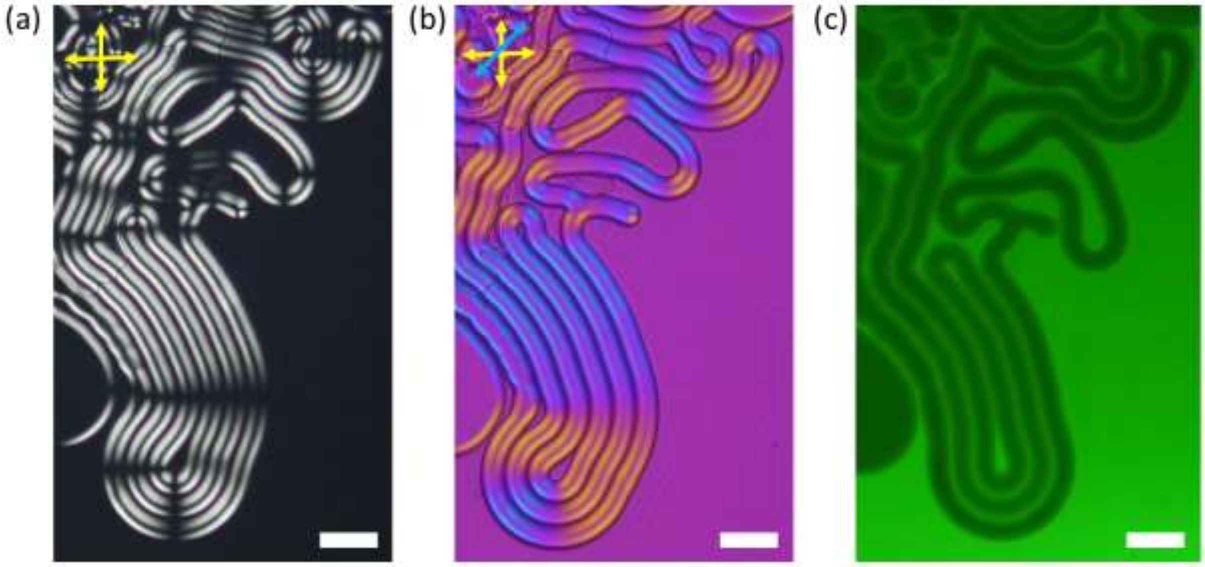

Figure S27. The image of MFs with fluorescein marker in water phase taken by polarized light microscope (a) without and (b) with the full-wavelength retardation plate. (c) Fluorescence image of the corresponding area of the sample ($\lambda_{exc.}$ = 460-495 nm). The scale bars represent 25 µm.

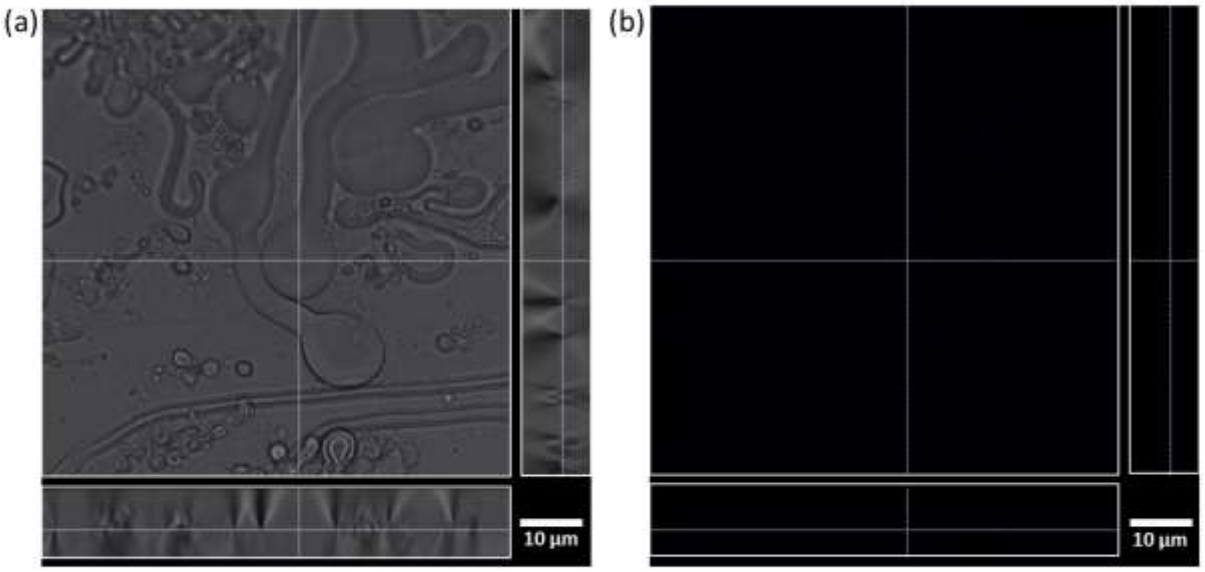

Figure S28. (a) The bright-field image of the MFs without the FA CNDs. (b) The fluorescence confocal image of the same area of the sample. Z-profiles are taken along white lines in the planes perpendicular to the x-y plane. Images were collected from the emission wavelength in a range from 410 to 480 nm at the excitation wavelength of 405 nm. The images were taken under the same conditions as the micrographs shown in **Fig. 4d**. Scale bars represent 10 µm.

Experimental setup for two-photon microscopy

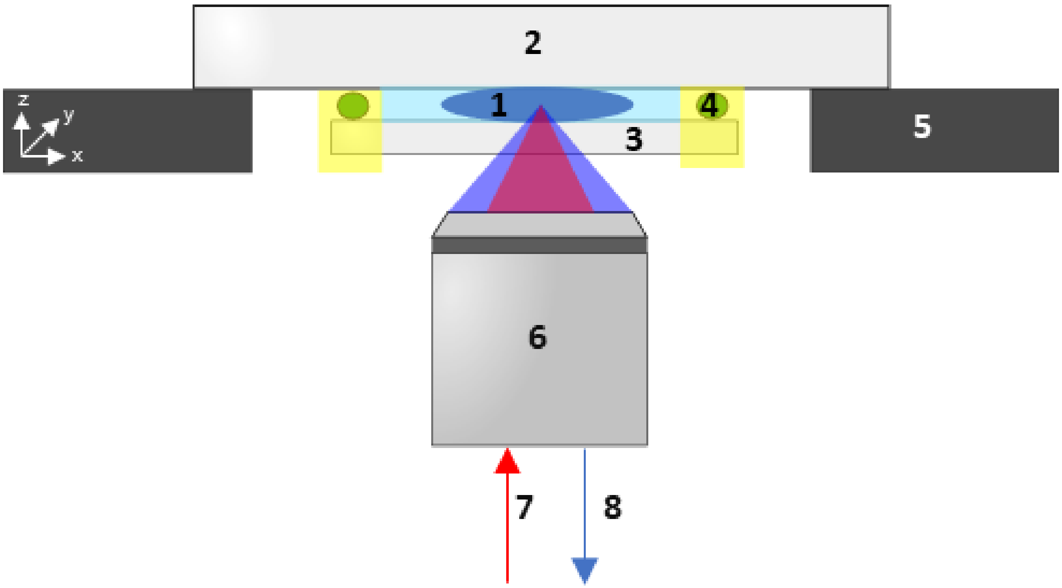

Figure S29. Schematic illustration of the liquid crystalline cell observed under an inverted epifluorescence microscope. (1) The hydrated lipid droplet is sandwiched between (2) the microscope slides and (3) the coverslip separated with (4) spacers immersed in glue. The sample is placed on (5) the XYZ positioning stage. (6) The objective focuses (7) the excitation laser beam and collects (8) the fluorescence emitted by the specimen. The unwanted traces of light are blocked by a dichroic mirror and optical filters (not shown).

Two photon-excited fluorescence imaging of MFs

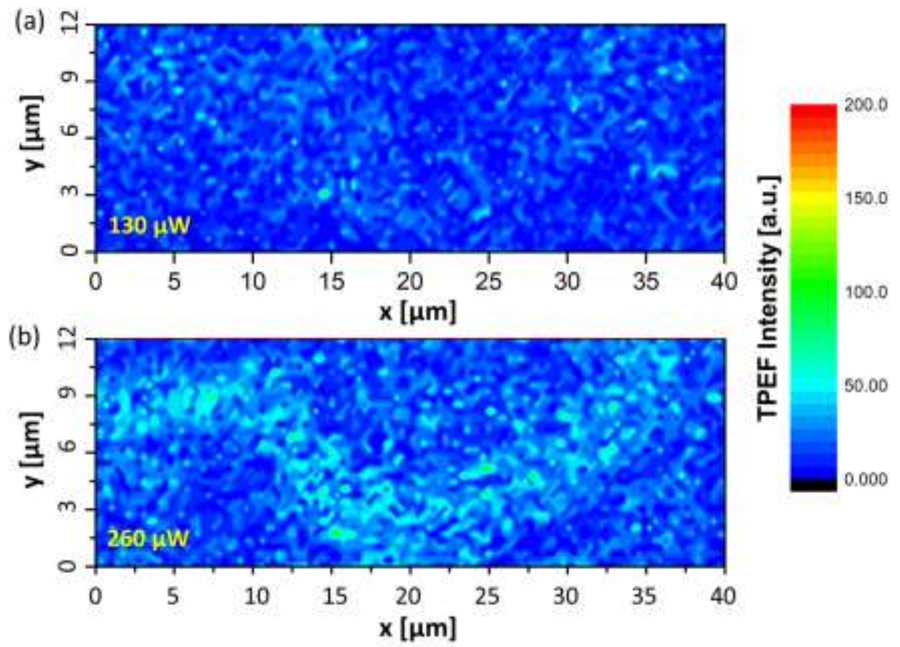

Figure S30. TPEF intensity images of the MFs without dopants excited at 740 nm with an average power of (a) 130 µW and (b) 260 µW.

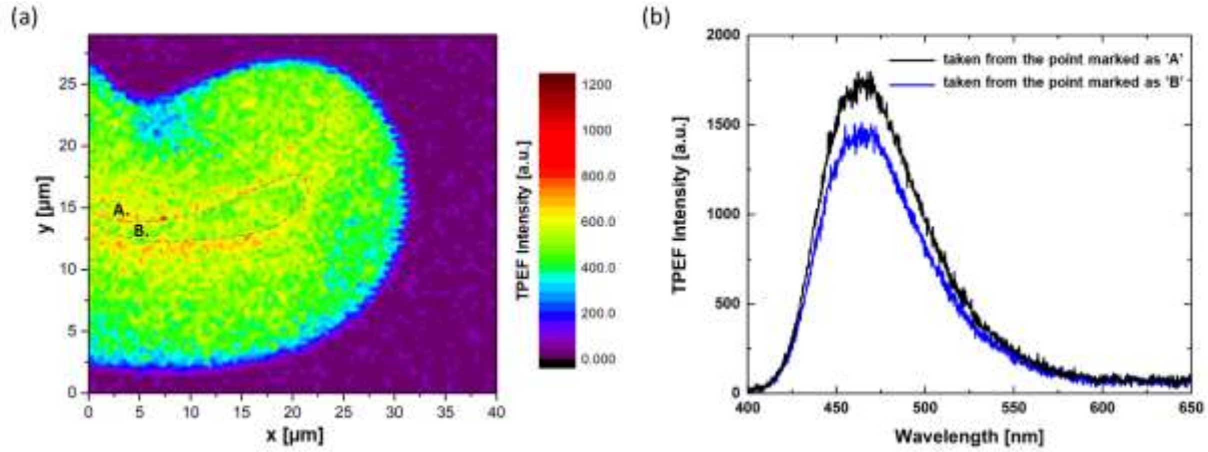

Figure S31. (a) The TPEF intensity map of the MFs doped with the FA CNDs. (b) The TPEF spectra of the FA CNDs within the DLPC matrix ($\lambda_{exc.}$ = 740 nm). The spectra were taken at the spot marked as 'A' (black line) and 'B' (blue line) in (a).

References:

1. Socrates, G. In *Infrared and Raman characteristic group frequencies : tables and charts*, 2001.
2. Castillo, J. J.; Rindzevicius, T.; Rozo, C. E.; Boisen, A., Adsorption and Vibrational Study of Folic Acid on Gold Nanopillar Structures Using Surface-Enhanced Raman Scattering Spectroscopy. *Nanomaterials and Nanotechnology* **2015,** *5*, 29.
3. Castillo, J. J.; Rindzevicius, T.; Wu, K.; Rozo, C. E.; Schmidt, M. S.; Boisen, A., Silver-capped silicon nanopillar platforms for adsorption studies of folic acid using surface enhanced Raman spectroscopy and density functional theory. *Journal of Raman Spectroscopy* **2015,** *46* (11), 1087-1094.
4. Tyagi, A.; Penzkofer, A., Fluorescence spectroscopic behaviour of folic acid. *Chemical Physics* **2010,** *367* (2), 83-92.
5. Thomas, A. H.; Lorente, C.; Capparelli, A. L.; Pokhrel, M. R.; Braun, A. M.; Oliveros, E., Fluorescence of pterin, 6-formylpterin, 6-carboxypterin and folic acid in aqueous solution: pH effects. *Photochemical & Photobiological Sciences* **2002,** *1* (6), 421-426.
6. Liu, H.; Li, Z.; Sun, Y.; Geng, X.; Hu, Y.; Meng, H.; Ge, J.; Qu, L., Synthesis of Luminescent Carbon Dots with Ultrahigh Quantum Yield and Inherent Folate Receptor-Positive Cancer Cell Targetability. *Scientific Reports* **2018,** *8* (1), 1086.
7. Wang, S.; Liu, S.; Zhang, J.; Cao, Y., Highly fluorescent nitrogen-doped carbon dots for the determination and the differentiation of the rare earth element ions. *Talanta* **2019,** *198*, 501-509.
8. Bakier, Y. M.; Ghali, M.; Sami, M.; Zahra, W. K., Highly luminescent un-doped carbon nano-dots driven from folic acid and passivated by polyethylene glycol. *Materials Today: Proceedings* **2020,** *33*, 1800-1803.
9. Bhunia, S. K.; Maity, A. R.; Nandi, S.; Stepensky, D.; Jelinek, R., Imaging Cancer Cells Expressing the Folate Receptor with Carbon Dots Produced from Folic Acid. *ChemBioChem* **2016,** *17* (7), 614-619.
10. Sarkar, P.; Ghosh, S.; Sarkar, K., Folic acid based carbon dot functionalized stearic acid-g-polyethyleneimine amphiphilic nanomicelle: Targeted drug delivery and imaging for triple negative breast cancer. *Colloids and Surfaces B: Biointerfaces* **2021,** *197*, 111382.
11. Campos, B. B.; Oliva, M. M.; Contreras-Cáceres, R.; Rodriguez-Castellón, E.; Jiménez-Jiménez, J.; da Silva, J. C.; Algarra, M., Carbon dots on based folic acid coated with PAMAM dendrimer as platform for Pt(IV) detection. *Journal of colloid and interface science* **2016,** *465*, 165-73.
12. Phan, L. M. T.; Gul, A. R.; Le, T. N.; Kim, M. W.; Kailasa, S. K.; Oh, K. T.; Park, T. J., One-pot synthesis of carbon dots with intrinsic folic acid for synergistic imaging-guided photothermal therapy of prostate cancer cells. *Biomaterials Science* **2019,** *7* (12), 5187-5196.
13. Makarov, N. S.; Drobizhev, M.; Rebane, A., Two-photon absorption standards in the 550–1600 nm excitation wavelength range. *Opt. Express* **2008,** *16* (6), 4029-4047.
14. Nyk, M.; Szeremeta, J.; Wawrzynczyk, D.; Samoc, M., Enhancement of Two-Photon Absorption Cross Section in CdSe Quantum Rods. *The Journal of Physical Chemistry C* **2014,** *118* (31), 17914-17921.
15. Nyk, M.; Wawrzynczyk, D.; Szeremeta, J.; Samoc, M., Spectrally resolved size-dependent third-order nonlinear optical properties of colloidal CdSe quantum dots. *Applied Physics Letters* **2012,** *100* (4), 041102.
16. Szeremeta, J.; Nyk, M.; Wawrzynczyk, D.; Samoc, M., Wavelength dependence of nonlinear optical properties of colloidal CdS quantum dots. *Nanoscale* **2013,** *5* (6), 2388-2393.
17. Wawrzynczyk, D.; Szeremeta, J.; Samoc, M.; Nyk, M., Optical nonlinearities of colloidal InP@ZnS core-shell quantum dots probed by Z-scan and two-photon excited emission. *APL Materials* **2015,** *3* (11), 116108.
18. Zaręba, J. K.; Nyk, M.; Samoć, M., Nonlinear Optical Pigments. Two-Photon Absorption in Crosslinked Conjugated Polymers and Prospects for Remote Nonlinear Optical Thermometry. *Polymers* **2020,** *12* (8), 1670.
19. Grynkiewicz, G.; Poenie, M.; Tsien, R. Y., A new generation of Ca2+ indicators with greatly improved fluorescence properties. *The Journal of biological chemistry* **1985,** *260* (6), 3440-50.

20. Gryczynski, I.; Lakowicz, J. R., Fluorescence intensity and anisotropy decays of the DNA stain Hoechst 33342 resulting from one-photon and two-photon excitation. *Journal of fluorescence* **1994,** *4* (4), 331-6.
21. Xu, C.; Webb, W. W., Measurement of two-photon excitation cross sections of molecular fluorophores with data from 690 to 1050 nm. *J. Opt. Soc. Am. B* **1996,** *13* (3), 481-491.
